# Correcting for Extreme Response Style: Model Choice Matters

**DOI:** 10.1177/00131644231155838

**Published:** 2023-02-17

**Authors:** Martijn Schoenmakers, Jesper Tijmstra, Jeroen Vermunt, Maria Bolsinova

**Affiliations:** 1 Tilburg University, The Netherlands

**Keywords:** item response theory, multidimensional nominal response model, IRTree model, response styles, extreme responding

## Abstract

Extreme response style (ERS), the tendency of participants to select extreme item categories regardless of the item content, has frequently been found to decrease the validity of Likert-type questionnaire results. For this reason, various item response theory (IRT) models have been proposed to model ERS and correct for it. Comparisons of these models are however rare in the literature, especially in the context of cross-cultural comparisons, where ERS is even more relevant due to cultural differences between groups. To remedy this issue, the current article examines two frequently used IRT models that can be estimated using standard software: a multidimensional nominal response model (MNRM) and a IRTree model. Studying conceptual differences between these models reveals that they differ substantially in their conceptualization of ERS. These differences result in different category probabilities between the models. To evaluate the impact of these differences in a multigroup context, a simulation study is conducted. Our results show that when the groups differ in their average ERS, the IRTree model and MNRM can drastically differ in their conclusions about the size and presence of differences in the substantive trait between these groups. An empirical example is given and implications for the future use of both models and the conceptualization of ERS are discussed.

Likert-type scales are frequently used in social science questionnaires to measure a wide array of constructs (Nemoto & Beglar, 2014; Van Vaerenbergh & Thomas, 2013; Willits et al., 2016). While the use of Likert-type scales is widespread, several threats to their valid use exist. Response style, the tendency of participants to provide a particular response to a question regardless of the questions content, is one of these threats (Falk & Cai, 2016; Van Vaerenbergh & Thomas, 2013). Response styles affect means and variances of Likert-type scales, thus potentially leading to unwarranted conclusions being drawn. For example, Moors (2012) found that the correlation between gender and leadership preference disappeared when response styles were taken into account. Due to the effects of response styles, it is important to detect and correct for response styles when conducting questionnaire research.

Many response styles exist, for example, acquiescent response style (ARS; tendency to agree with items regardless of content), mild response style (MLRS; tendency to avoid the scale endpoints), and extreme response style (ERS; tendency to respond using scale endpoints; Moors, 2012; Van Vaerenbergh & Thomas, 2013). While all of these response styles have the potential to reduce the validity of results based on Likert-type scales by affecting means and variances, response styles become especially relevant in contexts where different groups who may not deal with items in the exact same way are compared, such as in cross-cultural research. For this reason, response styles and their relation to culture have received extensive attention. In particular, many studies have shown ERS to differ substantially across cultures (Batchelor & Miao, 2016; Chun et al., 1974; Clarke, 2001; Hui & Triandis, 1989; Morren et al., 2011; Van Vaerenbergh & Thomas, 2013). The following section briefly summarizes antecedents and consequences of ERS.

ERS is defined as the tendency of participants to prefer responding extremely on item scales, independent of item content (Greenleaf, 1992; Van Vaerenbergh & Thomas, 2013). A variety of potential causes for ERS have been identified in the literature. These causes can be divided into questionnaire properties and person properties. Concerning the questionnaire, the use of bipolar scales and the way scale endpoints are labeled have been found to influence ERS in participants (Lau, 2007; Moors et al., 2014). Furthermore, the visual distance between scale options and whether options are presented horizontally or vertically has been shown to relate to ERS (Weijters et al., 2021). On the personal level, personality constructs such as neuroticism (Iwawaki & Zax, 1969), extraversion, and conscientiousness (Austin et al., 2006) influence ERS. In addition, race, gender, intelligence (Batchelor & Miao, 2016), and culture (Batchelor & Miao, 2016; Hui & Triandis, 1989; Morren et al., 2011) have been found to correlate with extreme responding.

As a response style, ERS has the potential to bias results obtained via questionnaires. Generally, ERS can reduce the magnitude of effects obtained, as extreme responding increases the variance of questionnaire responses (Van Vaerenbergh & Thomas, 2013). As an example, one study found that failing to account for ERS reduced explained variance from 69.5% to 53.5% (Lau, 2007). Given this potential distortion of results caused by ERS, various item response theory (IRT) models have been developed to detect and correct for ERS. The way these various models function and the assumptions they make regarding response styles differ substantially. One of the most notable differences between these models is conceptualizing ERS as a categorical versus continuous trait.

When conceptualizing ERS as a categorical latent trait, mixture IRT models can be used. Mixture IRT models are a combination of IRT modeling and latent class analysis, creating several latent classes based on observed responses (Rost, 1991). For every latent class, person and item parameters are estimated separately. Thus, there can be between-class differences in parameters, but within-class homogeneity is assumed. For example, a two-class model may result in one ordinary responding class and one extreme responding class (Austin et al., 2006; Bockenholt & Meiser, 2017). For modeling ERS, the mixture partial credit model is often applied (Cho, 2013; Huang, 2016; Sen & Cohen, 2019). While the categorical view of ERS is sometimes utilized in modeling, assuming a lack of within-class variation is not theoretically justified (Huang, 2016). Individuals within a class may very well differ in the presence and strength of their ERS tendency. As categorical ERS models are not able to model this within-class variation, this article will focus on models that model response styles continuously rather than categorically.

A variety of models that conceptualize ERS as a continuous variable have been developed. For example, extensions of the rating scale model (Jin & Wang, 2014), unfolding models (Javaras & Ripley, 2007), recent applications of multidimensional nominal response models (Bolt et al., 2014; Bolt & Johnson, 2009; Falk & Cai, 2016), item response tree (IRTree) models (Bockenholt, 2012; Bockenholt & Meiser, 2017; Meiser et al., 2019; Thissen-Roe & Thissen, 2013) and heterogeneous threshold models (Johnson, 2003) have been proposed. All of these models introduce an additional continuous latent variable for ERS in addition to the substantive latent variable that the questionnaire is intended to measure, but the models differ in the exact way in which the ERS dimension is included in the model.

While all these models allow one to correct for ERS, many of them do not have implementations in standard software packages. The lack of implementation in standard software packages makes models less likely to be used, especially by applied researchers. In addition, not all of these models have extensions beyond ERS to other response styles, and not all models are able to estimate a correlation between ERS and the substantive trait, making them less flexible. Two notable exceptions are the multidimensional nominal response model (MNRM) and the IRTree models which can be implemented in many standard packages for multidimensional IRT, for example, the R package mirt (Chalmers, 2012), are widely used (Zhang & Wang, 2020), and can be applied to a variety of response styles while estimating a correlation between the substantive trait and the response style(s) (Falk & Cai, 2016; Meiser et al., 2019; Zhang & Wang, 2020).

While both the MNRM and IRTree can flexibly be used to correct for ERS, the literature comparing these models in their ability to deal with response styles and their assumptions is sparse. Two notable exceptions that do compare MNRM and IRTree models should be discussed (Leventhal, 2019; Zhang & Wang, 2020). While the two aforementioned articles do compare MNRM and IRTree models, their parametrization and outcome measures differ substantially from the current article. Zhang and Wang (2020) focus on empirical rather than simulated data, limiting their conclusions somewhat, as true parameter values for the substantive and response style factors are unknown. In addition, both Leventhal (2019) and Zhang and Wang (2020) use a somewhat different operationalization of the MNRM and a substantially different IRTree model. Specifically, Zhang and Wang (2020) do not utilize a multidimensional node IRTree model. While Leventhal (2019) does utilize a multidimensional node IRTree model, they use a restrictive parameterization which reduces the validity of conclusions for less restrictive models. This issue will be discussed further in the Models section. Finally, neither article considers a multigroup setting. Multigroup settings are relevant in response style modeling, as there is an abundance of research linking response style to group characteristics such as culture (Batchelor & Miao, 2016; Chun et al., 1974; Clarke, 2001; Hui & Triandis, 1989; Morren et al., 2011; Van Vaerenbergh & Thomas, 2013). For this reason, the current article aims to expand upon previous research by comparing the MNRM and a multidimensional node IRTree model with minimal restrictions on their assumptions and ways of modeling ERS in a multigroup setting.

The rest of this article is organized as follows. First, the “Models” section presents the MNRM and IRTree models used in this article in detail and discusses the conceptual differences between these models. Second, the “Methods” section discusses the conditions of a simulation study that examines the practical impact of these conceptual differences in a multigroup context. Third, the “Results” section contains the results of the simulation study. Fourth, the “Empirical example” section shows that similar differences between the models as we observe in the simulation study can occur when the models are fit to real data. Finally, the article ends with a discussion where the conceptual and practical implications of the results for the future use of both models and the conceptualization of ERS are discussed.

## Models

The first model we discuss is the MNRM (Takane & de Leeuw, 1987). In the MNRM, the probability of endorsing an item category is the following:

(1) $$P\left(Y_{i}=k|\theta \right)=\frac{\exp\left({\tilde{\alpha }}_{\mathrm{ik}}^{T}\theta +c_{\mathrm{ik}}\right)}{\sum_{j=1}^{K}\exp\left({\tilde{\alpha }}_{\mathrm{ij}}^{T}\theta +c_{\mathrm{ij}}\right)},$$

where ${\tilde{\alpha }}_{\mathrm{ik}}$ is a vector of slope parameters, θ is a vector containing the participant’s scores on the latent traits, with the first element being the participants score on the substantive trait and the second element being the participant’s score on the ERS trait, and $c_{\mathrm{ik}}$ is a category intercept. Subscript *i* refers to items, and subscript *k* refers to categories (ranging from 1 to the number of categories). The MNRM has been used extensively to model response styles as continuous latent traits (Bolt et al., 2014; Bolt & Johnson, 2009; Bolt & Newton, 2011). Building on earlier work by Bolt and Johnson (2009), Falk and Cai (2016) developed a version of the MNRM that splits the vector of slope parameters ${\tilde{\alpha }}_{\mathrm{ik}}$ into an estimated item slope and a prespecified scoring matrix reflecting the loading of the response style(s) on categories:

(2) $$P\left(Y_{i}=k|\theta \right)=\frac{\exp\left({\left[a_{i}⊙s_{k}\right]}^{T}\theta +c_{\mathrm{ik}}\right)}{\sum_{j=1}^{K}\exp\left({\left[a_{i}⊙s_{j}\right]}^{T}\theta +c_{\mathrm{ij}}\right)},$$

where $a_{i}$ is a vector of item slope parameters, ⊙ denotes Schur/Hadamard multiplication, and $s_{k}$ is a vector of scoring matrix s . Due to splitting the slopes into a scoring matrix and an item slope, this model is able to flexibly model a variety of response styles while maintaining item-specific discrimination, unlike the model utilized in both Zhang and Wang (2020) and Leventhal (2019). Note that the models illustrated in this article will be limited to four-category items for simplicity and to reduce simulation time, although fewer or more categories are also possible under both models. While the formulation mentioned above uses item intercepts, these can easily be converted to item thresholds. For example, in the unidimensional case with a four-category item with three thresholds, the thresholds can be calculated as

(3) $$\tau_{g}=\frac{c_{\mathrm{ig}}-c_{i(g+1)}}{\alpha_{i}},$$

where $\tau_{g}$ denotes the *g*^th^ threshold, with *g* ranging from one to three. The conversion of intercepts to thresholds results in a somewhat more intuitive interpretation of parameters (the point on the substantive dimension where the *k*^th^ category is exactly as likely as the (*k*+1)^th^ category when the ERS is equal to 0). For this reason, the current paper will largely discuss item thresholds rather than item intercepts.

To illustrate the MNRM adaptation further, two examples of scoring matrices for a four-category item will be given. To specify the generalized partial credit model (i.e., no response styles) for a 4-category item, we could specify the scoring matrix as follows:

(4) $$[\begin{array}{cccc} 0 & 1 & 2 & 3 \end{array}].$$

Adding ERS, the scoring matrix would be

(5) $$\left[\begin{array}{cccc} 0 & 1 & 2 & 3\\ 1 & 0 & 0 & 1 \end{array}\right].$$

Note that many response styles could potentially be added to this model, but the current article will be limited to ERS.

**Figure 1.**
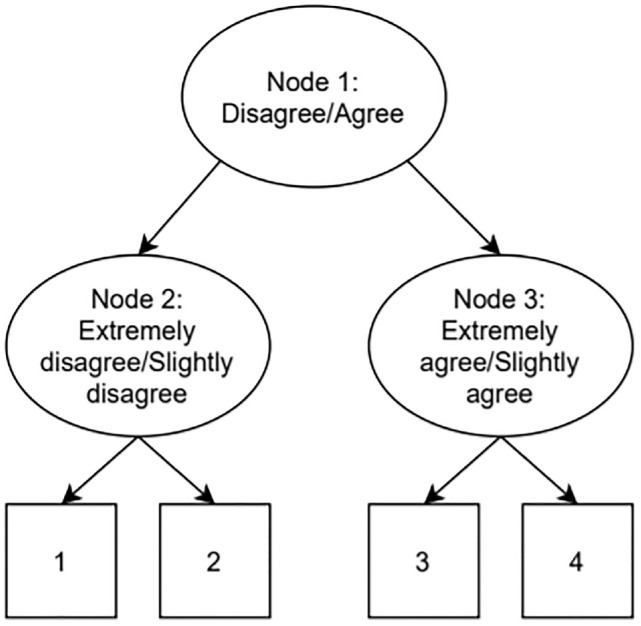
Example of an Item Response Tree Decision Process for a 4-Category Item.

Another class of widely used models for response styles that can be easily implemented in standard software are IRTree models (Bockenholt, 2012; Bockenholt & Meiser, 2017; Thissen-Roe & Thissen, 2013; Zhang & Wang, 2020). IRTree models are sequential decision models, where the response of a participant to a question is divided into multiple steps. An example of an IRTree four-category item decision process is illustrated in Figure 1.

The IRTree model depicted in Figure 1 splits the decision process up into multiple binary decision nodes, which can be modeled as pseudo-items. In effect, 1 four-category item is thus split up into 3 two-category pseudo-items. In the first node, a participant decides whether they agree or disagree with the four-category item. After this, depending on their decision in Node 1, they proceed to Node 2 or Node 3, where it is established if they agree/disagree moderately or extremely. Note that the sequential nature of this model does not necessarily imply a chronological order (Bockenholt & Meiser, 2017). While this type of IRTree is often used when modeling the effect of ERS on four-category items, a wide variety of IRTree models can be constructed based on a priori beliefs about a participants response process, the number of categories, the presence or absence of response styles, and so on (Bockenholt & Meiser, 2017).

Various parametrizations of the IRTree nodes have been proposed. Early examples of IRTree models modeled ERS by having the substantive dimension load on the first node and having the latent ERS dimension load on Node 2 and Node 3 (Bockenholt, 2012; Bockenholt & Meiser, 2017; Meiser et al., 2019). More recently, multidimensional IRTree models have emerged, where the substantive dimension loads on all three nodes and the latent ERS dimension loads on Node 2 and Node 3 (Meiser et al., 2019; Thissen-Roe & Thissen, 2013). This is unlike the IRTree models utilized in Zhang and Wang (2020), and the IRTree models mentioned before (Bockenholt, 2012; Bockenholt & Meiser, 2017), which only have the substantive dimension load on the first node. Having the substantive dimension load on all nodes rather than just the first node is desirable, as the substantive trait should conceptually also have a role to play in choosing between 1 or 2 and 3 or 4, rather than just influencing general agreement or disagreement. The desired multidimensional node IRTree model can be achieved by utilizing a unidimensional two-parameter logistic model for the first node and multidimensional IRT models for the second and the third node. To illustrate the desired multidimensional node model, let us by $Y_{\mathrm{im}}$ denote the response on the pseudo-item in node *m*. Table 1 depicts the relationship between the observed response on item *i* and the corresponding pseudo-items. Note that Nodes 2 and 3 are coded such that scores of 1 on these nodes correspond to higher scores for the observed response.

**Table 1.** Relationship Between the Observed Responses $Y_{i}$ and the Pseudoitem Responses $Y_{i1}$ , $Y_{i2}$ , and $Y_{i3}$ .

	Observed responses			
Pseudo-items	1	2	3	4
$Y_{i1}$ : Agreement	0	0	1	1
$Y_{i2}$ : Extreme/nonextreme disagreement	0	1	NA	NA
$Y_{i3}$ : Extreme/nonextreme agreement	NA	NA	0	1

Equation 6 depicts the general parameterization of the IRTree nodes:

(6) $$P\left(Y_{\mathrm{im}}=1|\theta \right)=\frac{\exp\left(\sum_{v=1}^{2}\alpha_{\mathrm{imv}}\theta_{v}\;+d_{\mathrm{im}}\right)}{1+\exp\left(\sum_{v=1}^{2}\alpha_{\mathrm{imv}}\theta_{v}+d_{\mathrm{im}}\right)},$$

where $\alpha_{\mathrm{imv}}$ denotes the slope parameter of item *i* in node *m* for dimension *v*, $\theta_{v}$ denotes the *v*^th^ latent trait, with the first dimension being the substantive trait and the second dimension being the ERS trait, and $d_{\mathrm{im}}$ denotes the intercept of item *i* in node *m*. In addition, several node-specific restrictions are present. In the first node, ERS has no effect, as participants are merely choosing whether they agree with an item or not, a process conceptually unrelated to ERS. For this reason, the slope $\alpha_{i12}$ is constrained to be equal to 0. In practice, a correlation between ERS and various substantive traits, such as personality and intelligence, is often found (Austin et al., 2006; Batchelor & Miao, 2016). However, without imposing any constraints on the item slopes, the correlation between the two dimensions is not identified. To allow for a correlation between ERS and the substantive trait to be modeled, $\alpha_{i21}$ is constrained to be equal to $\alpha_{i31}$ , and $\alpha_{i22}$ is constrained to be equal to $-\alpha_{i32}$ .

Note that the constraints that we use on the slope parameters result in a less restrictive IRTree model than the models used by Meiser et al. (2019) and Leventhal (2019). In Meiser et al. (2019), the slope parameters are constrained to be equal across items, which allows dropping the restriction of the slope in Node 2 being equal to minus the slope in Node 3 but still requires restricting the common slope in Node 2 to be of the opposite sign to the common slope in Node 3. In Leventhal (2019), a model originally proposed in Thissen-Roe and Thissen (2013) is used. This model has five rather than six item-specific parameters. In addition to constraining $\alpha_{i21}=\;\alpha_{i31}$ and $-\alpha_{i22}=\;\alpha_{i32}$ , a constraint $d_{i3}-d_{i2}=2d_{i1}\frac{\alpha_{i21}}{\alpha_{i11}}$ is imposed. As such, the difference in the intercepts in the third and second nodes is regressed on the intercept in the first node. This constraint is typically not used in other IRTree models, where the intercepts in all nodes are instead freely estimated (e.g., Bockenholt, 2012; Bockenholt & Meiser, 2017; Meiser et al., 2019). In our article, we chose a parametrization with item-specific slope parameters (i.e., more general than the constraints of Meiser et al., 2019) and node-specific intercepts (i.e., more general than the constraints of Leventhal, 2019).

While the MNRM and IRTree models are distinct in the way response styles are modeled, both utilize continuous latent traits for both the substantive and the response style dimensions. As the conceptualization of the latent traits seems quite similar, one could be led to believe the models would have close or identical results when utilized. However, the models also have some important differences. IRTree models split any single item up into multiple pseudo-items, while the MNRM utilizes a divide-by-total approach (the probability of a category is the numerator of that category divided by the summed numerators of all categories) to model all item categories in a single step. This seemingly small divergence results in major differences in the impact of ERS. The IRTree model’s separation of the item into pseudoitems allows for a very precise effect of ERS to be modeled. Specifically, as ERS loads on Nodes 2 and 3, but not Node 1, ERS only affects the probability of a response being extreme, without affecting the probability of general agreement with an item. At first glance, the scoring matrix of ERS in the MNRM, depicted in Equation 5, appears to achieve the same. However, the divide-by-total nature of the MNRM does not allow ERS to be modeled without affecting the agreement probability of items. This effect is most prominent for items that do not have thresholds symmetric around the substantive trait level of a participant (e.g., [−1, 0, 1] for $\theta_{1}=$ 0) but asymmetric thresholds (e.g., [−1, 0, 1] for $\theta_{1}=$ 1) when modeling ERS under the MNRM.

**Figure 2.**
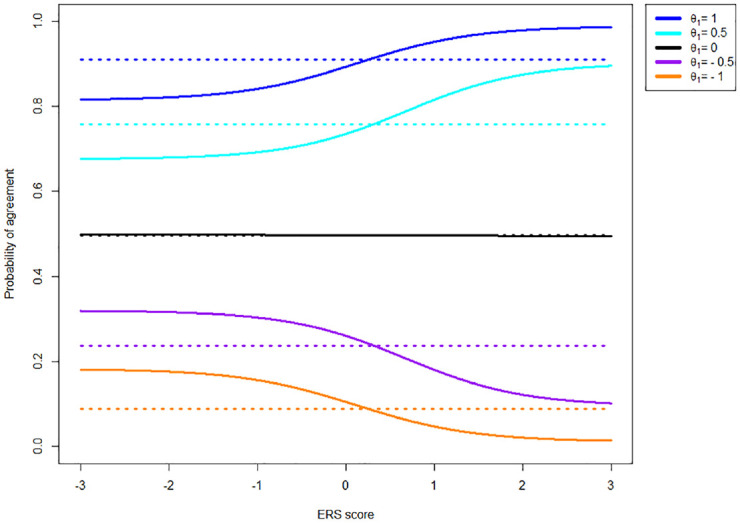
Probability of Agreement for an Item With Thresholds [−1, 0, 1] Given Various Levels of the Substantive Trait ( $\theta_{1})$ as a Function of ERS ( $\theta_{2})$ Under the MNRM (Solid Line) and IRTree Models (Dotted Line). *Note.* Colors indicate various $\theta_{1}$ values. ERS = Extreme response style; MNRM = multidimensional nominal response model; IRTree = item response tree.

To illustrate the difference between the models described above, several figures are provided. Data for the figures displayed here were generated using the MNRM, with a sample size of 50,000 participants with $\theta_{1}\sim N(0,1)$ , $\theta_{2}\sim N(0,1)$ , an α of 1.5 for both dimensions, the scoring matrix as depicted in Formula 5, and thresholds of −1, 0, 1. Note that these parameter values were chosen to match the simulation study described in the Methods section. The reasons for choosing these values are discussed in the Methods section and in Supplemental Appendix B. Item parameters for both the MNRM and the IRTree were estimated from the data using the mirt R package (Chalmers, 2012) and averaged over 10 identical items after which category probabilities were calculated. With the large sample size of 50,000 participants, parameter estimates showed very little uncertainty and were nearly identical to the true values. As figures using IRTree-generated data are very similar, these are displayed in Supplemental Appendix A. Figure 2 illustrates that the agreement probability depends on ERS under the MNRM but not under the IRTree, especially when the item thresholds are not symmetric around $\theta_{1}$ .

As can be seen in Figure 2, the probability of agreeing with an item under the IRTree never depends on the extent of ERS. Generally, the probability of agreeing under the MNRM does depend on ERS. The size of this effect varies depending on how symmetric the thresholds are around the substantive trail level.

**Figure 3.**
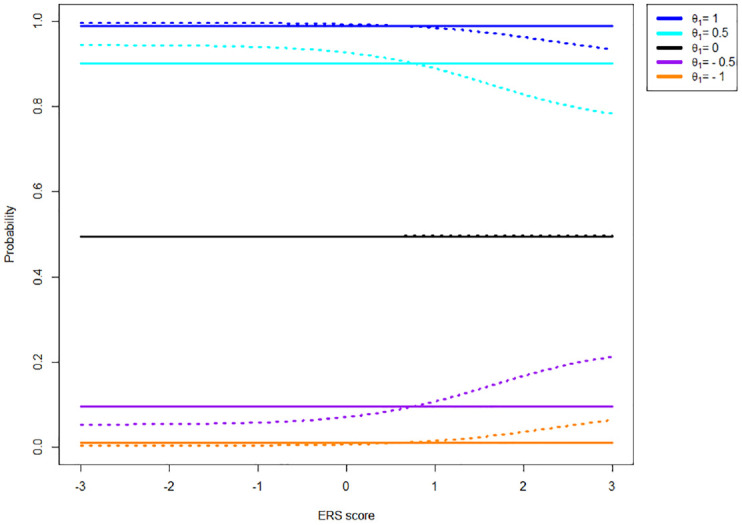
Probability of Endorsing Category Four of a Four-Category Item With Thresholds [−1, 0, 1] Given an Extreme Response and Various Levels of the Substantive Trait ( $\theta_{1})$ as a Function of ERS ( $\theta_{2})$ Under the MNRM (Solid Line) and IRTree (Dotted Line) Models. *Note.* Colors indicate various $\theta_{1}$ values. ERS = Extreme response style; MNRM = multidimensional nominal response model; IRTree = item response tree.

While the MNRM does not result in invariant agreement probability in the presence of ERS in combination with asymmetric item thresholds, it does offer a property not present in the IRTree model. Specifically, the probability of agreement given that the response is extreme (selecting a 4 versus selecting a 1) remains identical in the MNRM, regardless of the extent of ERS. This is not the case for the IRTree model, as is displayed in Figure 3.

As can be seen in Figure 3, the probability of endorsing Category 4 given an extreme response generally depends on the extent of ERS present in the data for the IRTree model but not for the MNRM model. Note that the same holds for the probability of agreement conditional on the response not being extreme (selecting a 3 vs. selecting a 2).

**Figure 4.**
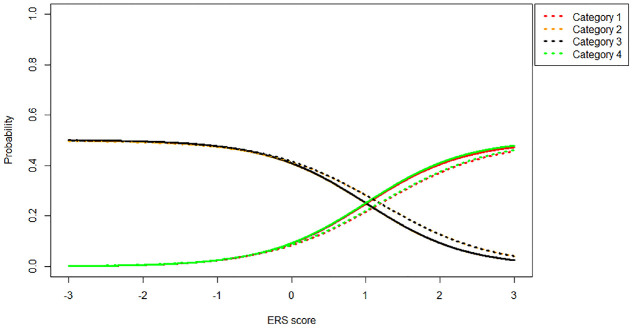
Category Probabilities (1 = Red, 2 = Orange, 3 = Black, 4 = Green) Under the MNRM (Solid Line) and IRTree (Dotted Line) Models for an Item With Symmetric Thresholds [−1, 0, 1] Given $\theta_{1}=0$ and Varying $\theta_{2}$ Values. *Note.* MNRM = multidimensional nominal response model; IRTree = item response tree; ERS = Extreme response style.

Overall, the two differences between the models discussed above result in different category probabilities for each model. Figure 4 shows the probabilities of different categories as a function of ERS when the substantive trait is kept constant at 0 (i.e., the item thresholds are symmetric around participants $\theta_{1}$ ). Solid lines indicate an MNRM model, while dotted lines indicate an IRTree model.

**Figure 5.**
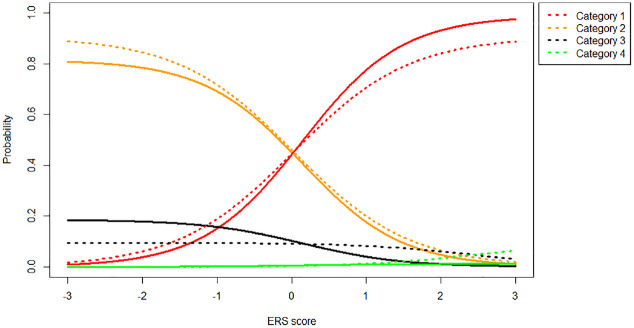
Category Probabilities (1 = Red, 2 = Orange, 3 = Black, 4 = Green) Under the MNRM (Solid Line) and IRTree (Dotted Line) Models for an Item With Asymmetric Thresholds [0, 1, 2] Given $\theta_{1}=0$ and Various $\theta_{2}$ Values. *Note.* MNRM = multidimensional nominal response model; IRTree = item response tree; ERS = Extreme response style.

As can be seen in Figure 4, the IRTree and MNRM models are quite close together when the item thresholds are symmetric around $\theta_{1}$ . As ERS increases, the probability of extreme responses (1 or 4) increases, and the probability of moderate responses (2 or 3) decreases, as one would expect. Figure 5 depicts the same scenario, but this time with an item with asymmetric thresholds (thresholds 0, 1, 2).

As we can see in Figure 5, the IRTree and MNRM models start to diverge more substantially when the item thresholds are not symmetric around $\theta_{1}$ and ERS is present. In this instance, we see the probability of endorsing category 1 increasing more rapidly for positive ERS values, and decreasing more rapidly for negative ERS values, under the MNRM than under the IRTree model. The probability of Category 2 decreases more for positive ERS values and decreases less for negative ERS values under the MNRM than under the IRTree. The difference between the two models is especially striking for Category 3. For the MNRM, the probability of Category 3 goes from ~0.2 at −3 ERS to ~0 at +3 ERS, while the IRTree probability remains almost constant at ~0.1, only shifting slightly downward at the higher ERS values. Finally, the probability of Category 4 remains close to 0 for the MNRM, regardless of ERS extent, while this probability starts to increase as ERS increases for the IRTree model.

While it is clear the MNRM and IRTree differ substantially in their modeling of response probabilities given ERS and items with asymmetric thresholds, the practical impact of these differences remains unclear. The differences between models may be especially relevant in a cross-cultural multigroup context, as culture has frequently been found to influence the extent to which individuals engage in ERS (Clarke, 2001; Johnson et al., 2005; Morren et al., 2011). To clarify the practical impact of using different models in a multigroup setting, a simulation study was conducted. The following section describes and explains the method chosen for this simulation.

## Method

To examine the practical impact of the conceptual differences between the MNRM and IRTree models discussed in the previous section, a simulation study was conducted. The bias in the substantive trait group mean and variance was of primary interest here. To generate the data required for the simulation study, R version 4.1.2 was used (R Core Team, 2017). First, MNRM data was generated. Second, data were generated using an IRTree model. Finally, a control condition (i.e., no ERS is present) was generated using a generalized partial credit model (GPCM). All code used to generate data and the generated data itself can be found at https://osf.io/nrgmy/.

### Multidimensional Nominal Response Model

To simulate a cross-cultural comparison, participants were divided in two groups. Participant substantive trait scores for both groups were drawn from N(0,1) , and ERS trait scores were drawn from N(0,1) for group 1, and N(μ,1) for group 2, where μ was 1, 0, or −1 depending on the condition. After careful consideration, which is further described in Supplemental Appendix B, we chose to use alphas of 1.5 for the both the substantive and the ERS dimension with item thresholds of [−1+m,0+m,1+m] or [0+m,1+m,2+m] depending on the condition, where m was added to all item thresholds to create varying item thresholds for different items as one would see in a test in practice. The m values were equally spaced between −0.5 and 0.5.

Together, these item loadings and item thresholds resulted in category probabilities that were not too low for any category (>.05), reasonable correlations between the latent traits and the item responses, and noticeable bias in the substantive trait mean and variance of group 2 when ignoring ERS (i.e., estimating a GPCM). The number of items (10 or 20) was also varied in the simulation. In total, this resulted in 2 (threshold sets) ×2 (number of items) ×3 (group 2 ERS means) =12 data generating conditions, with 500 replications per condition. All three models were applied to this data. Outcome measures were the bias in the substantive trait mean and variance in Group 2. For identification purposes, the substantive trait mean of Group 1 was fixed to 0, and the substantive trait variance in Group 1 was fixed to 1. As such, the mean of group 2 is the difference between the mean of Group 1 and Group 2, and the variance of Group 2 is the ratio of the variance in Group 2 to the variance in Group 1. Note that based on reviewer feedback, we also included sample size and distribution of the m-values as additional factors of the simulation. However, since the results were very similar to the main conditions, these additional results are presented in Supplemental Appendix E.

### Item Response Tree

For the IRTree model, person parameters were generated identically to the MNRM model. Item parameters were obtained by generating data under an MNRM with 500,000 participants per group, item parameters as described in the MNRM section and applying an IRTree model to these data to obtain IRTree item parameters estimates. These estimates were used as the true values for the IRTree item parameters when generating data to ensure maximum comparability between the IRTree and MNRM models. The same 12 conditions and the same outcome measures were used as for the MNRM.

### Control

In the control condition, no response style was present. To generate data with no response style present, the MNRM was used, with every participant scoring a 0 on the ERS dimension, equivalent to setting the item slope to 0 for the ERS dimension. In this case, the MNRM simplifies to a GPCM (Falk & Cai, 2016). Other item parameters were the same as in the MNRM. In the control condition, we varied the number of items in the test and which threshold was used, resulting in 2×2=4 conditions with the GPCM as the generating model. Outcome measures were the same as in the MNRM condition.

## Results

Results for the control condition with the GPCM as the data-generating model are displayed in Table 2. As the data were generated under the GPCM, no response style is present.

**Table 2.** Results for the Control Condition.

			MNRM		IRTree		GPCM	
Factors	$N_{\mathrm{items}}$	τ	$\mu_{\theta }$ bias	$\sigma^{2}$ bias	$\mu_{\theta }$ bias	$\sigma^{2}$ bias	$\mu_{\theta }$ bias	$\sigma^{2}$ bias
GPCM	10	[−1,0,1]	−0.002	0.005	−0.002	0.006	−0.002	0.006
		[0,1,2]	0.003	0.017	0.004	0.014	0.004	0.017
	20	[−1,0,1]	0.003	0.007	0.002	0.008	0.002	0.006
		[0,1,2]	0.001	0.006	0.002	0.004	0.002	0.005

*Note.* $\mu_{\theta }$ bias refers to bias in the substantive trait mean in group 2, $\sigma^{2}$ bias refers to bias in the substantive trait variance in Group 2, $N_{\mathrm{items}}$ refers to the number of items in the condition, and τ refers to the mean item thresholds. MNRM = multidimensional nominal response model; IRTree = item response tree; GPCM = generalized partial credit model.

As can be seen in Table 2, little bias occurs, regardless of which model is applied to the GPCM data. As there is almost no bias in any condition, the effect of factors is difficult to discern and is of little practical significance if present at all. Results seem to indicate that applying the MNRM or the IRTree model to data that has no response style present seems to have a negligible impact on the estimated substantive trait mean and variance. Table 3 displays the results when the MNRM is the data-generating model.

**Table 3.** Results for the MNRM Condition.

				MNRM		IRTree		GPCM	
Factors	ΔERS	$N_{\mathrm{items}}$	τ	$\mu_{\theta }$ bias	$\sigma^{2}$ bias	$\mu_{\theta }$ bias	$\sigma^{2}$ bias	$\mu_{\theta }$ bias	$\sigma^{2}$ bias
MNRM	−1	10	[−1,0,1]	0.000	0.015	−0.001	**−0.238**	−0.001	**−0.585**
			[0,1,2]	0.000	0.013	**0.147**	**−0.250**	**0.324**	**−0.541**
		20	[−1,0,1]	−0.003	0.006	−0.005	**−0.268**	−0.004	**−0.577**
			[0,1,2]	−0.005	0.022	**0.145**	**−0.265**	**0.317**	**−0.521**
	0	10	[−1,0,1]	0.002	0.008	0.000	0.007	0.000	0.005
			[0,1,2]	0.001	0.015	0.001	0.014	0.002	0.010
		20	[−1,0,1]	−0.001	0.022	−0.006	0.015	−0.003	0.009
			[0,1,2]	0.001	0.022	−0.001	0.016	−0.001	0.011
	1	10	[−1,0,1]	0.003	−0.002	0.002	**0.359**	0.003	**1.414**
			[0,1,2]	−0.002	0.019	**−0.192**	**0.401**	**−0.507**	**1.251**
		20	[−1,0,1]	0.001	0.040	−0.005	**0.419**	−0.006	**1.330**
			[0,1,2]	0.005	0.036	**−0.196**	**0.420**	**−0.471**	**1.108**

*Note.* $\mu_{\theta }$ bias refers to bias in the substantive trait mean in group 2, $\sigma^{2}$ bias refers to bias in the substantive trait variance in group 2, ΔERS refers to the difference in the ERS mean between group 1 (constant ERS mean at 0) and group 2 (−1, 0 or 1 ERS mean), $N_{\mathrm{items}}$ refers to the number of items in the condition, τ refers to the mean item thresholds. Values substantially differing from zero are marked in bold. MNRM = multidimensional nominal response model; IRTree = item response tree; GPCM = generalized partial credit model; ERS = Extreme response style.

Results for the MNRM-generated data shows substantially more bias than the GPCM-generated data. Several interesting trends emerge in these results. To start, the MNRM has no problems recovering the substantive trait mean and variance when it is the data-generating model. Both the GPCM and the IRTree do run into problems estimating these outcomes when data are generated under the MNRM, but only when the groups differ in ERS.

When the difference between the two groups mean ERS is −1, the variance in the substantive trait is underestimated by both the IRTree and GPCM, although for the GPCM underestimation is more severe than for the IRTree. Problems estimating the substantive trait mean only occur when an item threshold shift is introduced, creating asymmetry of the item thresholds around the mean participant substantive trait level. In this case, both the GPCM and IRTree overestimate the substantive trait mean, although the GPCM again does so more severely than the IRTree model.

As one may expect, these trends are reversed when the difference between the two groups ERS means is 1. In this case, both the IRTree and the GPCM overestimate the substantive trait variance when ERS mean difference between groups is present and underestimate the substantive trait mean when ERS mean difference between groups and item threshold shifts is present. In essence, the IRTree model seems to correct too little for the ERS present in the data when the MNRM is the generating model, reducing the bias compared with ignoring the response style completely but not eliminating it. Note that the extent to which the models show bias in both the substantive trait variance and the mean increases when the ERS mean in Group 2 is 1 compared to when it the ERS mean in group 2 was −1. This difference is small but noticeable for the IRTree but quite substantial in size for the GPCM.

Table 4 shows the same conditions as Table 3, but this time with the IRTree generating data. When the IRTree model generates data, it does not have problems estimating the substantive trait mean and variance in any condition. Just as before, the two models that did not generate data run into problems estimating substantive trait variance when the mean ERS difference between the two groups is not zero. This lack of group-level bias in the absence of ERS mean differences between the groups may lead one to believe that using a model to estimate the substantive trait other than the data-generating model does not lead to any substantive trait bias if there is no ERS mean difference between the groups. While this is true at the group level, individual-level results show bias occurring at the individual level even when groups do not differ in mean ERS (see Supplemental Appendices C and F).

**Table 4.** Results for the IRTree Condition.

				MNRM		IRTree		GPCM	
Factors	ΔERS	$N_{\mathrm{items}}$	τ	$\mu_{\theta }$ bias	$\sigma^{2}$ bias	$\mu_{\theta }$ bias	$\sigma^{2}$ bias	$\mu_{\theta }$ bias	$\sigma^{2}$ bias
IRTree	−1	10	[−1,0,1]	0.003	**0.354**	0.001	0.012	0.002	**−0.491**
			[0,1,2]	**−0.185**	**0.372**	−0.004	0.005	**0.259**	**−0.464**
		20	[−1,0,1]	−0.003	**0.386**	−0.004	0.011	−0.002	**−0.474**
			[0,1,2]	**−0.197**	**0.401**	−0.010	0.008	**0.248**	**−0.436**
	0	10	[−1,0,1]	0.001	0.017	0.000	0.015	0.002	0.015
			[0,1,2]	0.000	0.013	−0.001	0.011	−0.001	0.012
		20	[−1,0,1]	0.002	0.020	−0.002	0.012	0.001	0.009
			[0,1,2]	0.001	0.018	−0.002	0.012	−0.004	0.015
	1	10	[−1,0,1]	−0.003	**−0.271**	−0.002	0.008	−0.001	**0.899**
			[0,1,2]	**0.182**	**−0.295**	0.001	0.008	**−0.343**	**0.847**
		20	[−1,0,1]	−0.002	**−0.276**	0.000	0.008	−0.001	**0.799**
			[0,1,2]	**0.199**	**−0.285**	−0.001	0.010	**−0.323**	**0.744**

*Note.* Notation is as described above for the MNRM table. IRTree = item response tree; MNRM = multidimensional nominal response model; GPCM = generalized partial credit model.

Although the IRTree and GPCM showed bias in the same direction when the MNRM generated the data, this is not the case for the MNRM and GPCM with IRTree generated the data. When the ERS difference between the two groups is −1, the MNRM overestimates the substantive trait variance, while the GPCM underestimates it. After an item threshold shift is added, the MNRM underestimates the substantive trait mean, while the GPCM overestimates it. In both cases, the GPCM shows more bias in an absolute sense than the MNRM. Interestingly, the IRTree model appears to have less bias when estimating MNRM data than the MNRM has when estimating on IRTree data in these conditions. The reverse is true for the GPCM, which has less bias on MNRM data than on IRTree data.

When the ERS difference between the two groups is 1, the trends are again reversed. Now, the MNRM underestimates the substantive trait variance, while the GPCM overestimates it. After the threshold shift is introduced, the MNRM overestimates the substantive trait mean, while the GPCM underestimates it. Again, the MNRM has less bias in an absolute sense than the GPCM. While the GPCM suffers more bias when the difference between the groups’ mean ERS is positive than when it was negative, this is not unequivocally the case for the MNRM. In fact, the bias for the substantive trait variance seems to have decreased somewhat when the ERS mean difference between groups changed from negative to positive. The advantage the IRTree showed in estimating on MNRM data compared with the MNRM estimating on IRTree data in the negative mean ERS condition is also diminished, with the IRTree showing less bias for 20 items but more for 10 items. Essentially, the MNRM appears to overcorrect for the ERS present in the IRTree-generated data, reducing the bias somewhat compared with ignoring the response style but overshooting the mark and switching the sign of the bias in the process.

Summarizing, the importance of correcting for ERS using the true model becomes clear from the results. Ignoring the ERS present in the data by using a GPCM to estimate the substantive trait mean and variance leads to notable bias in the substantive trait variance when groups differ in mean ERS and leads to bias in the substantive trait mean when groups differ in ERS and asymmetrical thresholds are present. While the IRTree and the MNRM both do well in correcting ERS when they are the generating and estimating model, group differences in mean ERS and item threshold shifts again lead to bias when data are generated by the other model. Specifically, the IRTree model undercorrects for ERS in these conditions when the MNRM generates the data, reducing the bias in magnitude but failing to eliminate it. On the other hand, the MNRM overcorrects for ERS in these conditions when data is generated with an IRTree model, reducing the bias in magnitude but switching the sign of the bias. Thus, both models do not manage their goal of eliminating the effect of ERS on trait inference given group differences in mean ERS and item threshold shifts. As the results show the importance of selecting the right model to correct for ERS, tools for selecting the right model become very relevant. For this reason, an exploratory investigation into the use of model fit indices to select the right model for estimation is detailed in Supplemental Appendix D. While the results of this investigation naturally depend on the conditions considered, the use of the model fit indices for this purpose seems promising under the conditions studied in this paper.

## Empirical Example

To show that the differences between models found in the simulation study can also be found in real data, an empirical example is provided. To make this empirical example as similar as possible to the simulation study conducted in the paper, we looked for a multigroup dataset with items with four categories. As the Programme for International Student Assessment (PISA) is a well-known and publicly accessible source for multigroup data, we chose to look for this type of questionnaire here. We used the mathematics work ethic scale from PISA 2012 (Organisation for Economic Co-Operation and Development, 2013). The scale has 9 four-category items. Some examples of items in this scale were “I work hard on my mathematics homework” and “I pay attention in mathematics class. The response options were “Strongly disagree,”“Disagree,”“Agree,” and “Strongly agree,” and they were scored with higher scores indicating higher levels of mathematics work ethic.^1^ The mean test score across all countries was 2.86, indicating that the test scores are left skewed, which is important in illustrating the differences between the ERS models.

For the purposes of this example, we chose to examine Costa Rica (*N* = 2,863) and Malaysia (*N* = 3,389). These groups were preferred over other countries for several reasons. First of all, the countries substantially differed in mean ERS estimated by the MNRM (0.549 ERS mean difference between countries). Second, the countries were a good illustration of possible differences in conclusion between the IRT models, as models reached different conclusions regarding the substantive trait mean difference between Costa Rica and Malaysia. It is important to note here that we merely chose this example to show that it is possible to obtain different conclusions when utilizing different models; we do not claim that this will always (or often) happen in practice.

As a first step in the analysis, we estimated the GPCM, MNRM, and IRTree models with the mean and variance of the latent variables in Costa Rica fixed to 0 and 1 for identification purposes. The average item slopes under the MNRM were 1.235 for the substantive trait and 2.547 for the ERS trait, with average item thresholds equal to −3.263, −1.467, and 0.603, indicating asymmetric average item thresholds. Note that the ERS loading on the items and the asymmetry in item thresholds is stronger in the empirical example than in the simulation study, while the ERS mean difference between groups (0.549) is weaker than the ERS differences simulated.

Group-level results of Malaysia are displayed in Table 5. As can be seen in the table, conclusions regarding the substantive trait difference between Costa Ricans and Malaysians differ depending on the model used. Under the GPCM, the Malaysian group shows a significantly lower mean mathematics work ethic compared with the group from Costa Rica (95% confidence interval [ 95%CI]:[−0.417,−0.307]) . When modeling ERS using the IRTree, the difference in means between the countries decreases (95%CI:[−0.214,−0.077]) but remains significant. Under the MNRM, the countries do not differ significantly in mathematics work ethic (95%CI:[−0.107,0.057]) .

**Table 5.** Estimated Group Parameters in the Focal Group for the Various Models.

Model	$\mu_{\theta }$	$\mu_{\mathrm{ERS}}$	$\sigma_{\theta }^{2}$	$\sigma_{\theta ,\mathrm{ERS}}$	$\sigma_{\mathrm{ERS}}^{2}$
GPCM	−0.36(0.03)	NA	0.81(0.04)	NA	NA
MNRM	−0.03(0.04)	−0.55(0.04)	1.67(0.12)	−0.01(0.04)	1.08(0.08)
IRTree	−0.15(0.04)	−0.52(0.04)	1.35(0.08)	0.09(0.04)	1.11(0.08)

*Note.* $\mu_{\theta }$ denotes the substantive trait mean, $\mu_{\mathrm{ERS}}$ denotes the mean ERS, $\sigma_{\theta }^{2}$ is the substantive trait variance, $\sigma_{\theta ,\mathrm{ERS}}$ is the covariance between the substantive trait and ERS, $\sigma_{\mathrm{ERS}}^{2}$ is the ERS trait variance. GPCM = generalized partial credit model; MNRM = multidimensional nominal response model; IRTree = item response tree; ERS = Extreme response style.

Note that the differences between the estimated substantive trait mean between the models is of similar size to the differences found between models in the simulation study. It thus seems the increased asymmetry in item thresholds in combination with the higher ERS loading on items offset the lower ERS mean difference between groups in the empirical example. A final noteworthy result is that the MNRM correction for ERS also appears to be stronger here than the IRTree correction for ERS, which was also found in the simulation study. Overall, the results of the empirical example indicate substantive conclusions can differ not only depending on whether a correction for ERS is used but also which correction for ERS is used.

Table 6 contains the fit indices for each model. These results indicate that the GPCM is not the data-generating model, given the detected ERS by both models and the improved fit of both ERS models over the GPCM. Furthermore, the IRTree seems to exhibit the best fit. While one could choose to prefer the IRTree model on this basis, it may be more insightful to think about the conceptualization of ERS under different models when choosing which model to prefer as we elaborate on further in the discussion.

**Table 6.** Model Fit Indices for the Various Models.

Model	LogLik	Parameters	AIC	BIC	SABIC	HQ
GPCM	−51014.0	38	102,104.0	102,360.2	102,239.4	102,192.8
MNRM	−47846.6	51	95,795.3	96,139.1	95,977.0	95,915.4
IRTree	−47783.9	60	95,687.8	96,092.2	95,901.5	95,827.9

*Note.* LogLik = log-likelihood; AIC = Akaike information criterion; BIC = Bayesian information criterion; SABIC = Sample-size adjusted BIC; HQ = Hannan-Quinn information criterion; GPCM = generalized partial credit model; MNRM = multidimensional nominal response model; IRTree = item response tree.

## Discussion

The present study set out to compare two widely used and flexible IRT models in their modeling of ERS under a variety of conditions. First, conceptual differences between the two models were compared. Second, the practical implications of these differences were examined by means of a multigroup simulation study with the bias of the group mean and variance as outcome measures. The results will be discussed in this order.

Conceptually, the IRTree and MNRM models appear to be very different in their modeling of ERS. Beyond the obvious differences in the IRTree modeling the response process as a multistep process and the MNRM using a divide-by-all approach, two major less obvious differences between the models were found. First of all, it was revealed that under the MNRM, the extent of ERS influences the probability of agreeing with an item. This effect is particularly noticeable when item thresholds are not symmetric around the participant’s substantive trait level. In the IRTree model, the probability of agreeing (responding with 3 or 4) with the item is independent of ERS. As ERS is often conceptualized as influencing only the probability of an extreme response, not the probability of agreeing with an item, the IRTree model seems to fit this conceptualization better. However, under the IRTree model, the probability of agreeing with an item given an extreme response (i.e., responding with 1 vs. 4 on a four-category item) depends on the extent of ERS present. The same holds for the probability of agreeing with an item given a non-extreme response (i.e., responding with 2 vs. 3 on a four-category item). In contrast, under the MNRM these probabilities are independent of ERS. While both models thus technically have a property the other model does not have, we find it difficult to imagine a situation where the property of agreeing with an item given an extreme response is of primary concern over the unconditional probability of agreeing with an item. Of course, it is up to researchers to consider their conceptualization of ERS and which of these two properties they value more before deciding which model to use. If both properties are deemed to be of importance, other models not presented in this paper may be of interest, although these models may have other shortcomings in modeling ERS.

The practical impact of the differences between the two models was examined using a multigroup simulation study. Results indicate that completely ignoring the response style by using a GPCM leads to substantial bias in the estimated variance of Group 2 when groups differ in their mean ERS levels. If thresholds are not symmetric around the average substantive trait level and groups differ in mean ERS, the estimation of the mean substantive trait of Group 2 is also biased.

These results are somewhat surprising, as some previous research on the consequences of ignoring ERS found minimal effects on individual trait estimates, reliability, and the correlation between substantive traits as long as the response style and the substantive trait are uncorrelated (Plieninger, 2017; Wetzel et al., 2016). The fact that an effect of ERS is found here but not in previous research may be due to several reasons. First, the current article considers a multigroup context, whereas both Plieninger (2017) and Wetzel et al. (2016) considered only a single group. Second, the current article examines items with locations not centered around the mean of the substantive trait. This is an important scenario to consider, as many psychological questionnaires do not have symmetric thresholds (i.e., items have expected values not exactly at the middle of the scale; consequently, the mean of the test is not exactly in the middle of the theoretical test range). As one example of this, Big Five personality scores are often found to be quite far removed from the middle point of the scale (Soliño & Farizo, 2014; Weisberg et al., 2011). Another example of a test likely to be severely skewed is mental health tests such as the Beck depression inventory, especially when applied in a nonclinical population (Beck, 1961; Gorenstein et al., 2005). If groups with a different mean ERS were to be compared on their personality or mental health status using the wrong ERS model (or no ERS model at all), we could thus expect results to be biased both in mean and variance.

A second main result is that using an MNRM to estimate the substantive trait mean and variance of IRTree-generated data, or using an IRTree model to estimate the substantive trait mean and variance of MNRM-generated data, runs into problems in the same conditions as when the GPCM is used for estimation. While the bias resulting from using the “wrong” ERS model is smaller than the bias that results from ignoring the response style altogether, both the substantive trait variance and the substantive trait mean can be substantially biased if the model used for estimation is not the data-generating model. The size of the bias that can be expected when using the “wrong” model to correct for ERS, or when ignoring ERS completely, is mainly based on two factors. First, the presence of an ERS mean difference between the groups leads to differences between the IRTree and MNRM model in the substantive trait variance. While the present study only formally examined ERS mean differences of −1, 0 and 1, larger ERS mean differences between the groups are expected to lead to larger differences between models. Second, the combination of ERS mean differences between groups and asymmetric item thresholds leads to differences between the models in both the substantive trait mean and variance. While we again only formally examined two levels of item threshold asymmetry, we expect that the differences between the models will increase in size as item threshold asymmetry increases. One valuable insight here may be that asymmetric item thresholds necessarily lead to skewed test scores (i.e., mean test scores that are not exactly in the middle of the theoretical range of test scores). We thus recommend researchers who are interested in the possible bias resulting from applying the wrong model to the data to check the skewness of the test scores empirically.

A second note when comparing the models is that the IRTree model seems to undercorrect for ERS when the data-generating model is the MNRM, while the MNRM overcorrects for ERS when the data-generating model is the IRTree model. This is caused by the MNRM-generating data where the extent of ERS influences the probability of agreement, which the IRTree cannot model, and the IRTree-generating data where the probability of agreement given an extreme response depends on the extent of ERS, which the MNRM cannot model. These results point to the importance of choosing a model to estimate the substantive trait that is compatible with the model that created the data.

While the importance of picking the right model is clear, it is less clear how this can best be achieved. From one perspective, the difference between models presented here can be seen as a fundamental difference between the conceptualization of ERS between the MNRM and IRTree models. In the IRTree model, the ERS trait only becomes relevant after an initial decision between agreeing or not agreeing with the item is made in Node 1, while the MNRM assumes no such steps in the response process. From this perspective, it follows that the choice between models should be based on conceptual views of how ERS should function. Note that these conceptual views on ERS are not limited to choices between the models but also include choices on how to specify the models. For example, the scoring matrix in the MNRM used in this simulation study assumes symmetry in the ERS effect, which does not need to be the case, and other scoring matrices with asymmetric ERS effects could be specified. Similarly, the constraint of equal ERS loadings across Nodes 2 and 3 in the IRTree model assumes a symmetry in the ERS effect across nodes and can potentially be removed by imposing other constraints.

A second perspective on choosing the right model is that the model that empirically has the best fit to the dataset the researcher works with should be preferred. For this reason, an exploratory analysis of using fit indices for model selection was conducted (see Supplemental Appendix D). Results indicate that the use of the Akaike information criterion or log-likelihood (due to the low cost of preferring a more complex model over the GPCM), Bayesian information criterion (BIC), sample-size adjusted BIC, or Hannan-Quinn information criterion for model selection is promising. One avenue of further research could be the development of a tool that examines model fit based on the fundamental conceptual differences between the two models outlined earlier, rather than general model fit. Beyond picking the best model of the two models presented here, one may also question whether other models that are not presented here, or perhaps even models that do not yet exist, created the data they are currently examining. Future research would do well to further compare existing models, both conceptually and practically, test if current fit indices can be used to select the right models when other models are compared and develop new models with alternative conceptualizations of the response process. Finally, future research could work on making IRTree models more accessible for the applied researcher, as setting up a properly specified IRTree model is currently not the easiest of tasks.

Despite the current findings, this article also has some limitations. First of all, the paper is limited to ERS and only compares two models. As indicated earlier, the literature on ERS models alone is quite substantial, and more conceptual and practical differences may be uncovered by studying other models more in detail. On top of this, ERS is only one of many response styles. As large conceptual differences between ERS models were uncovered in this paper, it would not be surprising to see similar differences for other response styles such as midpoint responding, acquiescent responding, and so on. For these reasons, future research should aim to expand the framework presented here to other models and response styles. As a second limitation, the current article is based on a simulation study, which is naturally limited in how many conditions can be displayed and how realistic the conditions are compared to real data. For example, all item slopes were fixed to 1.5 in this study, and all items had equidistant thresholds, which is unlikely to occur in real data. Future research should examine if these results are the same when other parameters are used. Third, only items with four categories were included in this article. It is thus not guaranteed that results generalize to items with more categories, especially if a middle category is also present. Future studies should examine the effects discussed here on items with differing number of categories and a middle category. Finally, the substantive trait mean was not varied between groups, making it impossible to infer what happens when groups are not identical in their substantive trait.

From this article, several practical recommendations can be made. First of all, a researcher should take great care in considering which model to use when modeling ERS. In this consideration, a conceptual underpinning of the expected response process and the effects of ERS should take center stage. In addition, model fit indices for the various models should be consulted to pick a model for estimation that is as close to the data-generating model as possible.

Second, the importance of considering ERS in a context where groups may differ in their ERS propensity is illustrated. Using a GPCM on data that contains ERS can result in substantial bias in the substantive trait mean and variance. While using the “wrong” ERS model on the data does not completely resolve these issues, it at least seems to reduce the bias. While further research is needed to confirm that this holds for conditions and models not considered in this study, the preliminary data presented here suggests the use of an ERS model that is not the data-generating model when ERS is present is preferable to not acknowledging the presence of ERS at all.

Overall, this article reveals that the MNRM and IRTree models cannot be used interchangeably to correct for ERS. Conceptual differences between the models were examined, and the practical impact of these differences on both the group and the individual level was illustrated using a simulation study. Researchers would do well to consider these differences between the models and their impact on future research when attempting to correct for ERS.

## Supplemental Material

sj-pdf-1-epm-10.1177_00131644231155838 – Supplemental material for Correcting for Extreme Response Style: Model Choice Matters Click here for additional data file. Supplemental material, sj-pdf-1-epm-10.1177_00131644231155838 for Correcting for Extreme Response Style: Model Choice Matters by Martijn Schoenmakers, Jesper Tijmstra, Jeroen Vermunt and Maria Bolsinova in Educational and Psychological Measurement

## Notes

1. Note that in the original datafile, items were coded as 1 (*Strongly agree*), 2 (*Agree*), 3 (*Disagree*), and 4 (*Strongly disagree*). Items were recoded for the purposes of this analysis so that the latent variable can be interpreted as mathematics work ethic rather than a lack of mathematics work ethic.
