# Supplementary material for: Correcting for Extreme Response Style: Model Choice Matters
Source: Educ Psychol Meas. 2023 Feb 17;84(1):145–70. doi: 10.1177/00131644231155838 (PMC10795569; doi:10.1177/00131644231155838)
Supplement: sj-pdf-1-epm-10.1177_00131644231155838 – Supplemental material for Correcting for Extreme Response Style: Model Choice Matters [file sj-pdf-1-epm-10.1177_00131644231155838.pdf]

Appendix A: IRTree-generated plots of agreement probability, probability of category 4 given an extreme response, and category probabilities

Figure A1:

Probability of Agreement for an Item with Thresholds [-1, 0, 1] Given Various Levels of the Substantive Trait (θ_1) as a Function of ERS (θ_2) Under the MNRM and IRTree Models with the IRTree Model Generating Data.

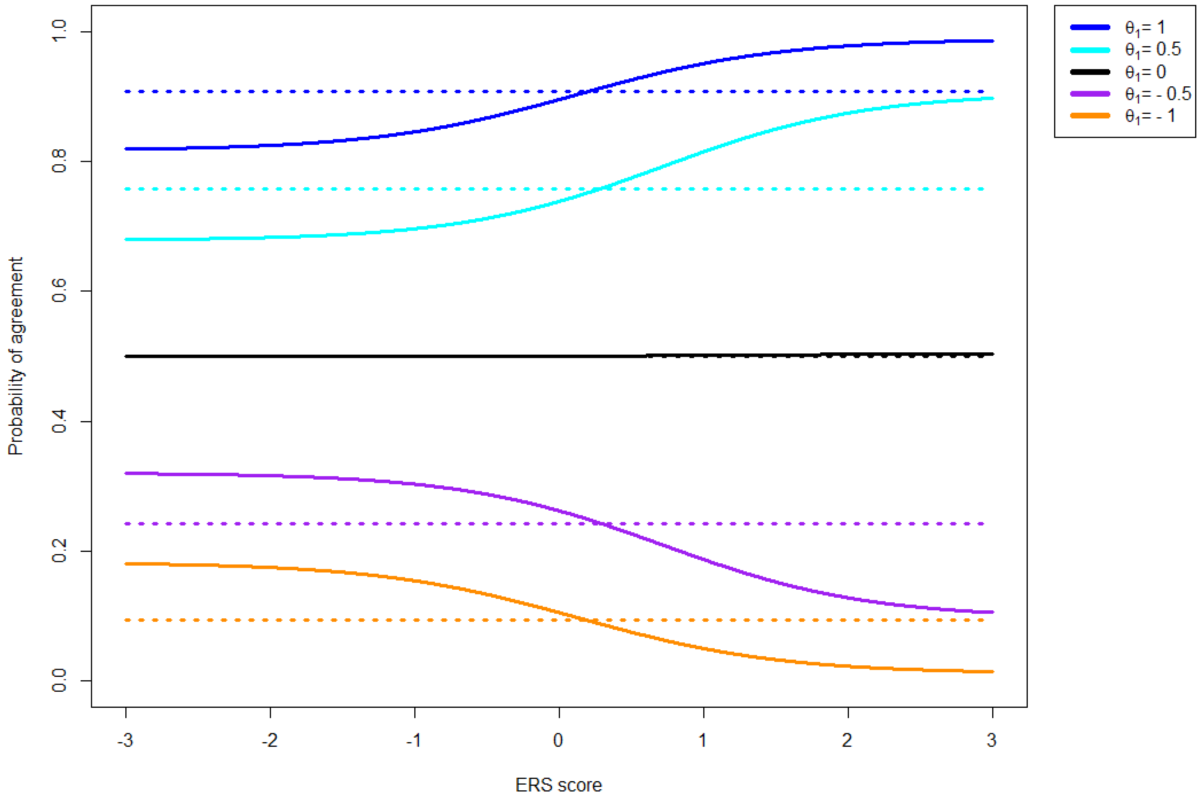

Figure A2:

Probability of Endorsing Category Four of a Four-category Item with Thresholds [-1, 0, 1] Given an Extreme Response for Various θ_1 and θ_2 Under the MNRM and IRTree Models with the IRTree Model Generating Data.

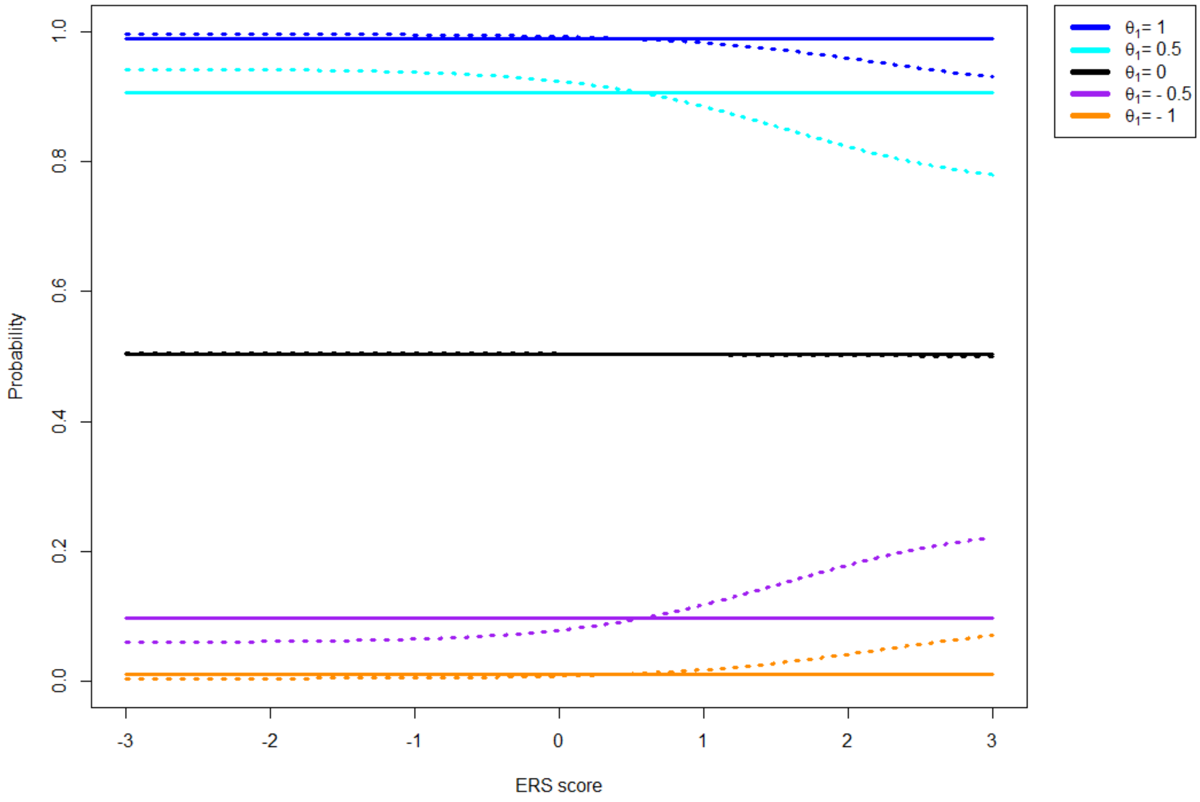

Figure A3:

Category Probabilities Under the MNRM and IRTree Models for an Item with Symmetric Thresholds [-1, 0, 1] Given $\theta_1 = 0$ and Varying θ_2 with the IRTree Model Generating Data.

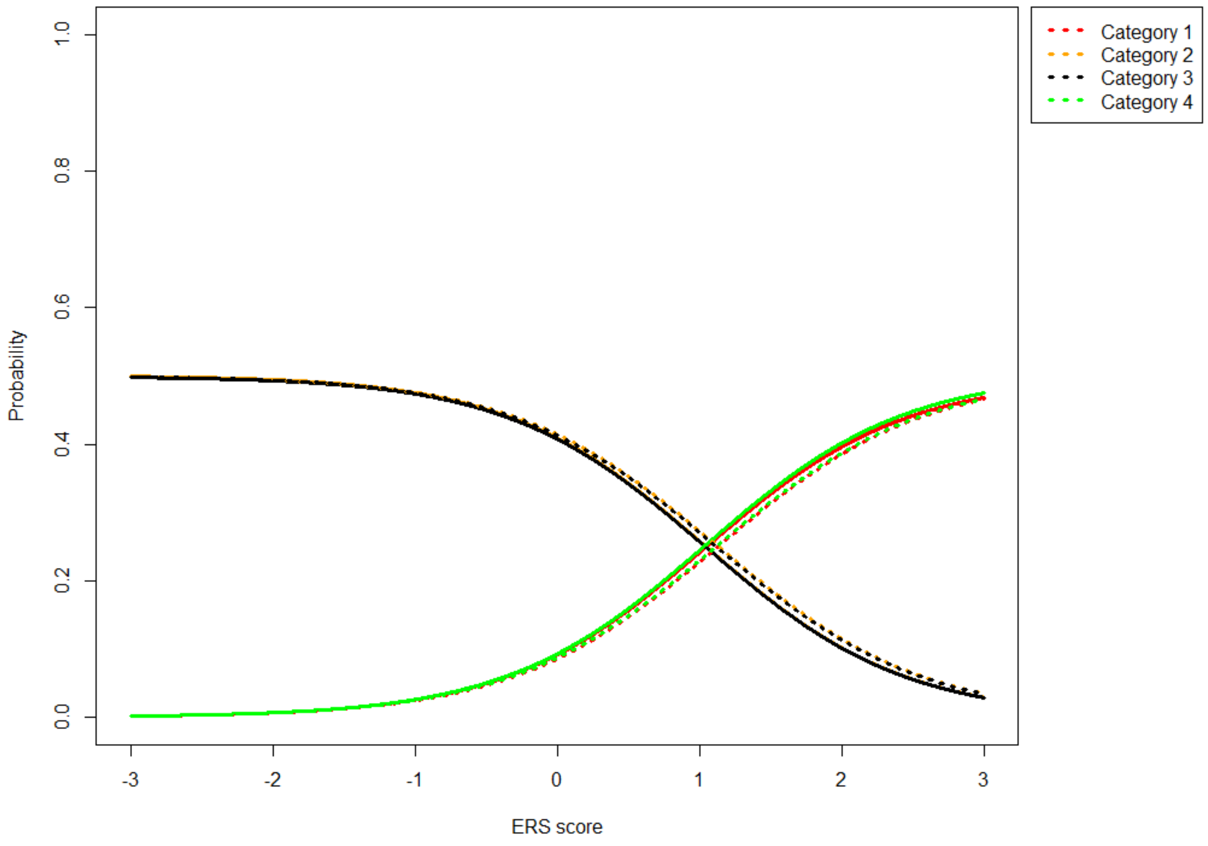

Figure A4:

Category Probabilities Under the MNRM and IRTree Models for an Item with Asymmetric Thresholds [0, 1, 2] Given $\theta_1 = 0$ *and Various* θ_2 *Values with the IRTree Model Generating Data.*

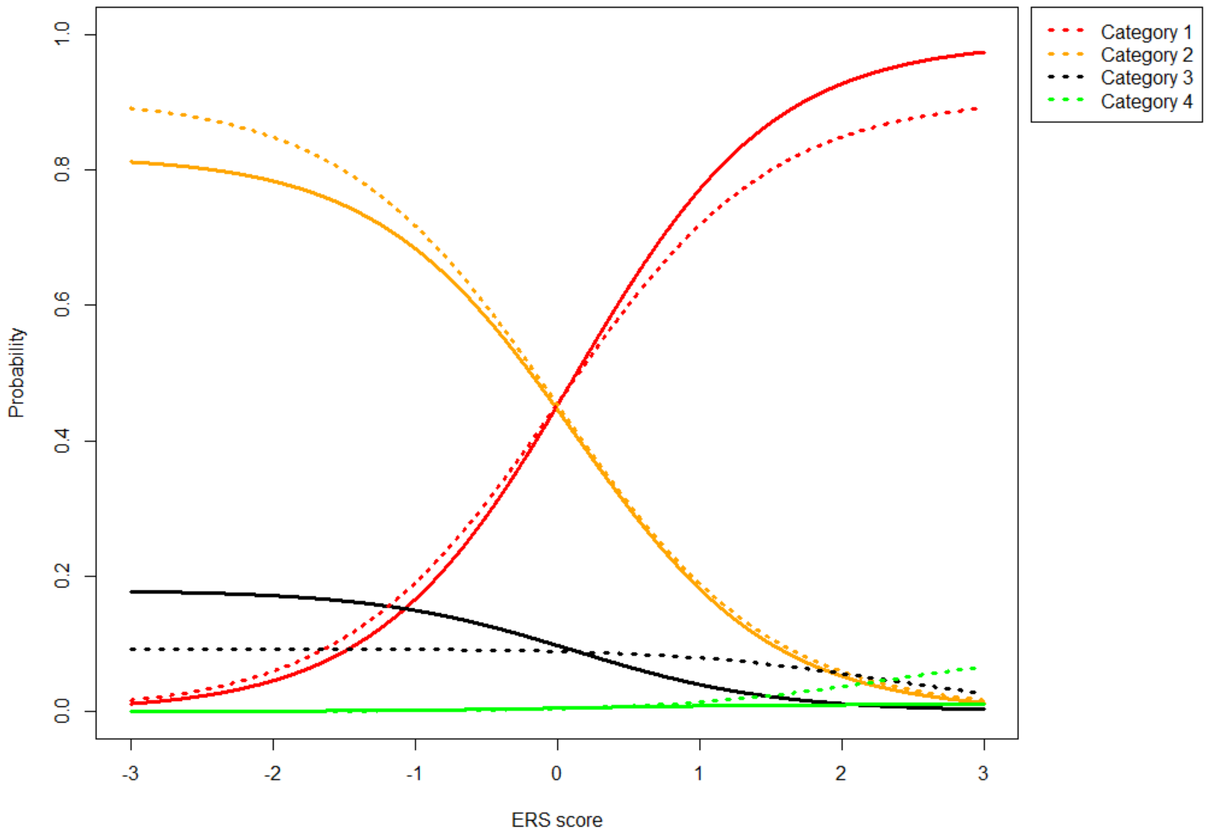

Appendix B: Details of the item parameter choice in the MNRM

In order to generate data, several item parameters were required. First of all, item thresholds were needed to model the overall item difficulty and the transition point between categories. Second, item slopes were necessary to model the relationship between the latent trait and the category probabilities. As was shown above, the effect of ERS on agreement probability depends on how symmetric the threshold is around participants substantive trait level. For this reason, we added a "threshold shift" as a final factor in the simulation study. The threshold shift is a constant which gets added to all thresholds in order to create asymmetry around participants' average substantive trait level in the item thresholds. For example, a thresholds shift of 1 would mean symmetrical thresholds [-1, 0, 1] get shifted to asymmetrical thresholds [0, 1, 2].

To choose the parameters, we conducted a small simulation study, considering various options for all parameters. Relevant outcomes were category probabilities for both groups, the correlation between the substantive trait and the observed response, the correlation between the extreme response trait and an extreme response, and the amount of bias in mean and variance of the substantive trait when a GPCM was applied to data generated with an MNRM with $ERS \sim N(0, 1)$ for group 1 and $ERS \sim N(1,1)$ for group 2. Results for all parameters considered are displayed below.

Table B1:

Exploratory Simulation Study of Various MNRM Parameters and Their Impact on Category Probabilities in Both Groups, the Correlation Between the Substantive Trait and the Observed Answers, the Correlation Between the ERS trait and an Extreme Answer, and Bias in the Mean and Variance when Estimating Using a GPCM.

τ	α_1, α_2	$\tau+$	$P(1)_1$	$P(2)_1$	$P(3)_1$	$P(4)_1$	$P(1)_2$	$P(2)_2$	$P(3)_2$	$P(4)_2$	$r_{x_i,\theta}$	$r_{E,ERS}$	θ bias	σ^2 bias
[-1, 0, 1]	1,1	0	0.208	0.291	0.292	0.209	0.308	0.195	0.193	0.304	0.611	0.388	-0.015	1.222
		0.5	0.310	0.325	0.237	0.128	0.434	0.211	0.160	0.195	0.602	0.383	-0.222	1.205
		1	0.417	0.333	0.179	0.071	0.562	0.210	0.118	0.110	0.571	0.377	-0.426	1.073
		1.5	0.530	0.314	0.120	0.036	0.680	0.187	0.078	0.055	0.523	0.365	-0.606	0.956
	1.5, 1.5	0	0.210	0.292	0.292	0.207	0.329	0.172	0.173	0.327	0.691	0.481	0.000	1.643
		0.5	0.324	0.331	0.225	0.120	0.478	0.190	0.137	0.196	0.680	0.476	-0.296	1.493
		1	0.448	0.335	0.155	0.062	0.622	0.180	0.093	0.105	0.637	0.456	-0.551	1.372
		1.5	0.575	0.305	0.094	0.027	0.743	0.152	0.056	0.048	0.577	0.438	-0.702	1.071
	2, 2	0	0.210	0.291	0.293	0.206	0.340	0.158	0.159	0.343	0.733	0.537	0.013	1.958
		0.5	0.331	0.337	0.217	0.116	0.498	0.173	0.124	0.205	0.717	0.528	-0.320	1.782
		1	0.464	0.338	0.142	0.056	0.651	0.163	0.082	0.104	0.671	0.507	-0.602	1.517
		1.5	0.596	0.301	0.080	0.023	0.774	0.135	0.047	0.045	0.605	0.480	-0.785	1.183

τ	α_1, α_2	$\tau+$	$P(1)_1$	$P(2)_1$	$P(3)_1$	$P(4)_1$	$P(1)_2$	$P(2)_2$	$P(3)_2$	$P(4)_2$	$r_{x_i,\theta}$	$r_{E,ERS}$	θ bias	σ^2 bias
	1, 1.5	0	0.213	0.287	0.286	0.214	0.338	0.161	0.160	0.340	0.601	0.508	0.003	1.791
		0.5	0.310	0.322	0.234	0.134	0.473	0.176	0.132	0.219	0.590	0.502	-0.318	1.730
		1	0.417	0.335	0.173	0.074	0.601	0.176	0.098	0.125	0.552	0.494	-0.576	1.533
		1.5	0.525	0.319	0.118	0.037	0.715	0.158	0.064	0.063	0.505	0.483	-0.781	1.303
	1.5, 1	0	0.203	0.298	0.297	0.202	0.294	0.206	0.207	0.293	0.705	0.361	0.002	1.073
		0.5	0.317	0.334	0.234	0.115	0.436	0.225	0.165	0.175	0.692	0.352	-0.205	1.039
		1	0.450	0.332	0.161	0.057	0.583	0.213	0.113	0.091	0.654	0.341	-0.388	0.934
		1.5	0.587	0.293	0.096	0.024	0.715	0.178	0.067	0.040	0.600	0.324	-0.504	0.717
[-1.5, 0, 1.5]	1,1	0	0.162	0.337	0.338	0.163	0.258	0.242	0.241	0.258	0.586	0.372	-0.001	1.296
		0.5	0.245	0.382	0.275	0.098	0.372	0.269	0.200	0.160	0.577	0.371	-0.234	1.234
		1	0.345	0.396	0.205	0.054	0.495	0.268	0.147	0.090	0.555	0.368	-0.430	1.103
		1.5	0.449	0.384	0.140	0.027	0.609	0.246	0.099	0.046	0.516	0.365	-0.581	0.940
	1.5, 1.5	0	0.153	0.347	0.347	0.153	0.268	0.231	0.231	0.270	0.668	0.458	0.003	1.611
		0.5	0.244	0.400	0.270	0.085	0.403	0.256	0.181	0.161	0.659	0.455	-0.282	1.537
		1	0.353	0.420	0.184	0.043	0.538	0.254	0.126	0.081	0.628	0.445	-0.521	1.279

τ	α_1, α_2	$\tau+$	$P(1)_1$	$P(2)_1$	$P(3)_1$	$P(4)_1$	$P(1)_2$	$P(2)_2$	$P(3)_2$	$P(4)_2$	$r_{x_i,\theta}$	$r_{E,ERS}$	θ bias	σ^2 bias
		1.5	0.473	0.395	0.113	0.019	0.668	0.221	0.075	0.037	0.582	0.441	-0.684	0.995
	2, 2	0	0.147	0.355	0.352	0.146	0.273	0.223	0.224	0.280	0.715	0.507	0.020	1.840
		0.5	0.242	0.418	0.262	0.078	0.418	0.249	0.171	0.161	0.700	0.502	-0.318	1.762
		1	0.355	0.437	0.172	0.037	0.566	0.244	0.111	0.079	0.662	0.492	-0.596	1.427
		1.5	0.483	0.406	0.097	0.015	0.695	0.208	0.063	0.034	0.610	0.485	-0.740	1.066
	1, 1.5	0	0.173	0.325	0.327	0.176	0.301	0.200	0.200	0.299	0.580	0.494	-0.014	1.861
		0.5	0.258	0.370	0.266	0.106	0.423	0.223	0.165	0.189	0.569	0.490	-0.314	1.717
		1	0.351	0.389	0.199	0.060	0.545	0.224	0.122	0.109	0.543	0.487	-0.581	1.546
		1.5	0.452	0.384	0.135	0.029	0.657	0.207	0.081	0.055	0.494	0.486	-0.768	1.289
	1.5, 1	0	0.141	0.359	0.360	0.140	0.225	0.276	0.277	0.223	0.676	0.333	-0.004	1.017
		0.5	0.231	0.413	0.279	0.077	0.348	0.307	0.216	0.129	0.668	0.334	-0.197	1.000
		1	0.345	0.428	0.191	0.036	0.484	0.301	0.150	0.065	0.640	0.324	-0.364	0.885
		1.5	0.474	0.395	0.116	0.015	0.623	0.261	0.089	0.027	0.602	0.324	-0.498	0.685
[-1.25, 0, 1.25]	1,1	0	0.185	0.314	0.315	0.186	0.283	0.219	0.218	0.280	0.599	0.383	-0.014	1.251
		0.5	0.279	0.353	0.255	0.113	0.406	0.238	0.178	0.178	0.592	0.380	-0.224	1.189

τ	α_1, α_2	$\tau+$	$P(1)_1$	$P(2)_1$	$P(3)_1$	$P(4)_1$	$P(1)_2$	$P(2)_2$	$P(3)_2$	$P(4)_2$	$r_{x_i,\theta}$	$r_{E,ERS}$	θ bias	σ^2 bias
		1	0.382	0.365	0.191	0.063	0.527	0.238	0.134	0.102	0.562	0.374	-0.417	1.097
		1.5	0.493	0.347	0.129	0.031	0.647	0.215	0.088	0.050	0.520	0.367	-0.581	0.919
	1.5, 1.5	0	0.178	0.322	0.322	0.179	0.296	0.202	0.203	0.299	0.681	0.470	0.005	1.596
		0.5	0.283	0.367	0.247	0.102	0.438	0.223	0.160	0.179	0.667	0.464	-0.277	1.535
		1	0.401	0.376	0.172	0.051	0.582	0.216	0.110	0.093	0.636	0.451	-0.523	1.279
		1.5	0.528	0.346	0.104	0.022	0.707	0.185	0.066	0.042	0.583	0.440	-0.675	0.997
	2, 2	0	0.176	0.323	0.325	0.176	0.310	0.189	0.190	0.311	0.723	0.528	0.006	1.885
		0.5	0.284	0.379	0.242	0.096	0.463	0.210	0.146	0.181	0.708	0.516	-0.340	1.787
		1	0.409	0.387	0.158	0.046	0.610	0.202	0.096	0.092	0.670	0.505	-0.597	1.453
		1.5	0.542	0.351	0.089	0.019	0.741	0.167	0.054	0.038	0.614	0.489	-0.758	1.060
	1, 1.5	0	0.193	0.305	0.307	0.195	0.319	0.181	0.182	0.319	0.591	0.502	-0.006	1.774
		0.5	0.286	0.347	0.249	0.118	0.447	0.199	0.149	0.205	0.579	0.497	-0.303	1.773
		1	0.386	0.362	0.185	0.067	0.573	0.200	0.110	0.117	0.549	0.492	-0.564	1.521
		1.5	0.487	0.353	0.127	0.033	0.687	0.182	0.072	0.060	0.499	0.486	-0.782	1.300
	1.5, 1	0	0.168	0.329	0.331	0.171	0.260	0.241	0.242	0.257	0.689	0.349	-0.013	1.063

τ	α_1, α_2	$\tau +$	$P(1)_1$	$P(2)_1$	$P(3)_1$	$P(4)_1$	$P(1)_2$	$P(2)_2$	$P(3)_2$	$P(4)_2$	$r_{x_i,\theta}$	$r_{E,ERS}$	θ bias	σ^2 bias
		0.5	0.275	0.373	0.258	0.094	0.394	0.265	0.192	0.149	0.682	0.344	-0.208	0.981
		1	0.397	0.379	0.177	0.046	0.536	0.256	0.131	0.077	0.649	0.335	-0.382	0.905
		1.5	0.530	0.342	0.108	0.020	0.669	0.219	0.078	0.034	0.604	0.327	-0.491	0.673

Note. This table is based on MNRM data, with θ_1 ~ N(0, 1) and θ_2 ~ N(0, 1) in group 1 and θ_1~ N(0, 1) and θ_2 ~ N(1,1) in group 2. Various values of intercepts and slopes were chosen to observe effect on probabilities of categories and correlations between traits and responses. τ is the value of the thresholds, $\tau +$ is the extent of the thresholds shift, P(1)1 is category probability 1 for group 1, P(2)1 is category probability 2 for group 1, P(1)2 is category probability 1 for group 2, etc. , $r_{x_i,\theta}$ is the correlation between substantive trait and item response, $r_{E,ERS}$ is the correlation between ERS trait and an extreme response, θ bias is the bias that occurs in the substantive trait mean when the GPCM is ran on the MNRM data, and σ^2 bias is the bias that occurs in the substantive trait variance when the GPCM is ran on the MNRM data.

Based on the results in Table B1, we decided to use thresholds of [-1, 0, 1], alphas of 1.5 and a threshold shift of 1. We chose these values to make sure no category probability in either group was below 0.05. In addition, we aimed for a correlation between the substantive trait and the observed response of 0.6, and a correlation between the ERS trait and the extreme response of around 0.

Appendix C: Individual level results in the absence of ERS mean differences between the groups

To illustrate the presence of individual level bias even when the two groups have identical ERS means, two plots are presented. As every replication in the simulation study had unique person parameters, it is not possible to look at bias for a single individual. Instead, participants with similar substantive trait levels are binned per condition across all replications in bins of size 0.2 ranging from -2 to +2 on the substantive trait. After, the average difference between the estimate of the substantive trait and its true value per bin is calculated. This gives an indication of substantive trait bias at the individual level for that range of substantive trait values. As the number of items did not have a substantial impact on the mean substantive trait bias per bin, results are presented for 20 item conditions only. In addition, only participants from group 2 are included to prevent possible estimated differences between groups from influencing results. Figure C1 presents the results when the MNRM generates data and no ERS mean difference between groups is present. Note that solid lines represent item thresholds of [-1, 0, 1], while dotted lines indicate item thresholds of [0, 1, 2]. Black lines indicate GPCM individual trait estimates, red lines indicate MNRM individual trait estimates, and blue lines indicate IRTree individual trait estimates.

Figure C1

Individual Substantive Trait Bias when the MRNM is the Generating Model and No ERS Differences Between Groups are Present

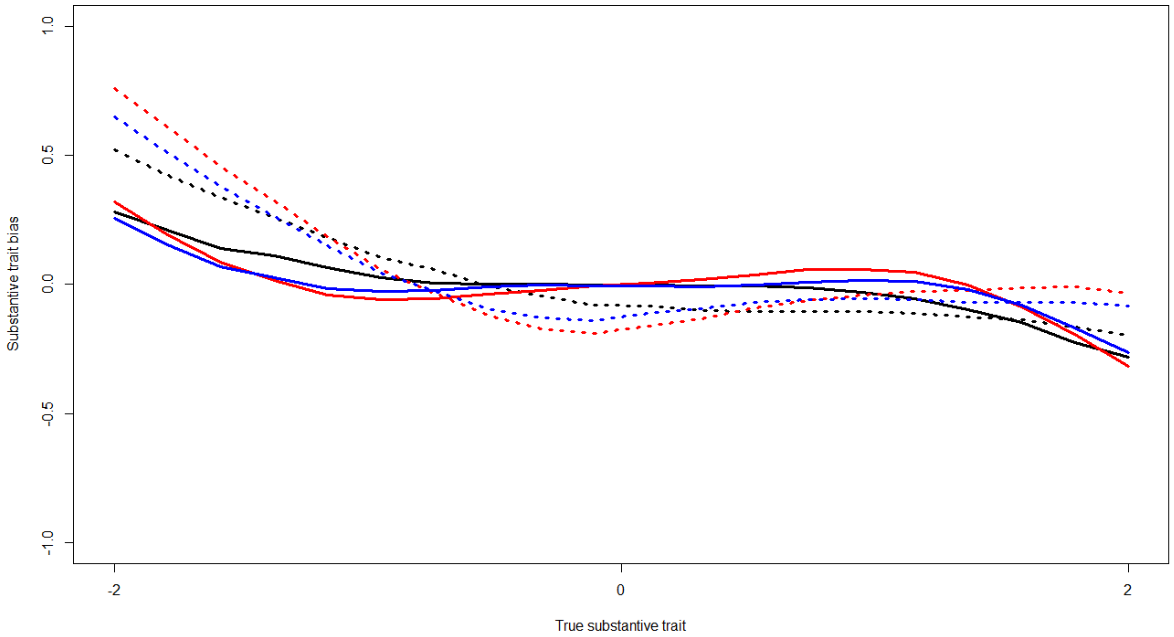

As Figure C1 shows, the GPCM differs quite a bit from both the IRTree and the MNRM in its individual substantive trait estimate bias, both when thresholds of [-1, 0, 1] and thresholds of [0, 1, 2] are used. While the IRTree and MNRM models differ less, some differences are still visible, especially at the extreme ends of the true substantive trait spectrum. Figure C2 presents the same graph, but this time with the IRTree model generating data.

Figure C2

Individual Substantive Trait Bias when the IRTree is the Generating Model and No ERS Differences Between Groups are Present

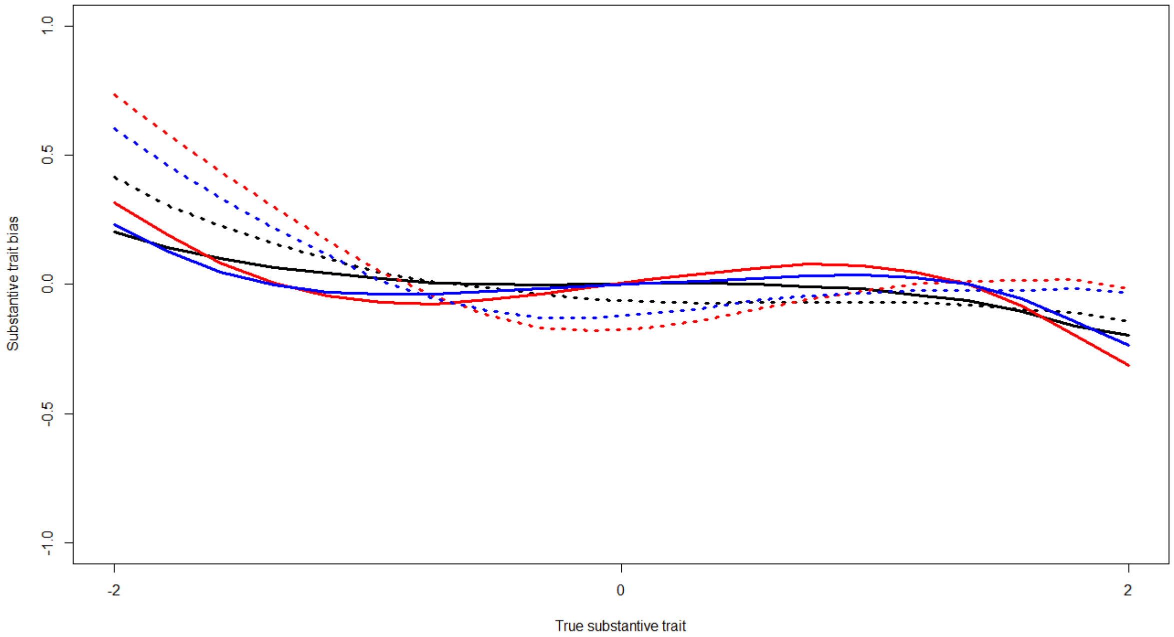

Figure C2 presents a similar trend as Figure C1. Again, GPCM estimates are substantially removed in individual substantive trait bias from both the IRTree and MRNM. While the IRTree and MRNM are closer, differences are still visible, especially at the extremes of the true substantive trait values. These two graphs together stress the point that the lack of group-level substantive trait bias in the absence of ERS mean differences between the groups does not translate to an absence of substantive trait bias at the individual level. Even when groups do not differ in mean ERS, model choice should still be considered carefully if one is interested in individual trait estimates in any way.

Appendix D: Do model fit indices select the right model?

Table D1:

Model Fit Indices Model Selection Frequencies When the GPCM is the Generating Model

N_{items}	τ	AIC			BIC			SABIC			HQ			LogLik		
		MNRM	Tree	GPCM	MNRM	Tree	GPCM	MNRM	Tree	GPCM	MNRM	Tree	GPCM	MNRM	Tree	GPCM
10	$[-1,0,1]$	8	337	155	0	0	500	0	3	497	0	3	497	33	467	0
	$[0,1,2]$	10	324	166	0	0	500	0	8	492	0	6	494	43	457	0
20	$[-1,0,1]$	7	399	94	0	0	500	0	1	499	0	0	500	37	463	0
	$[0,1,2]$	8	344	148	0	0	500	0	4	496	0	3	497	31	469	0

Table D1 displays the model selection frequencies for several fit indices when the GPCM is the generating model. In the table, we see the log-likelihood and the AIC are somewhat liberal in this condition, often selecting the more complicated models over the GPCM model. The BIC performs perfectly, always selecting the GPCM. The SABIC and HQ both perform very well, although not quite perfectly. Table D2 displays the model selection frequency when the MNRM is the generating model.

Table D2:

Model Fit Indices Model Selection Frequencies When the MNRM is the Generating Model

ΔERS	N_{items}	τ	AIC			BIC			SABIC			HQ			LogLik		
			MNRM	Tree	GPCM	MNRM	Tree	GPCM	MNRM	Tree	GPCM	MNRM	Tree	GPCM	MNRM	Tree	GPCM
-1	10	$[-1,0,1]$	499	1	0	499	1	0	499	1	0	499	1	0	499	1	0
		$[0,1,2]$	495	5	0	495	5	0	495	5	0	495	5	0	495	5	0
	20	$[-1,0,1]$	500	0	0	500	0	0	500	0	0	500	0	0	500	0	0
		$[0,1,2]$	499	1	0	499	1	0	499	1	0	499	1	0	499	1	0
0	10	$[-1,0,1]$	496	4	0	496	4	0	496	4	0	496	4	0	496	4	0
		$[0,1,2]$	494	6	0	494	6	0	494	6	0	494	6	0	494	6	0
	20	$[-1,0,1]$	500	0	0	500	0	0	500	0	0	500	0	0	500	0	0
		$[0,1,2]$	500	0	0	500	0	0	500	0	0	500	0	0	500	0	0
1	10	$[-1,0,1]$	499	1	0	499	1	0	499	1	0	499	1	0	499	1	0
		$[0,1,2]$	499	1	0	499	1	0	499	1	0	499	1	0	499	1	0
	20	$[-1,0,1]$	500	0	0	500	0	0	500	0	0	500	0	0	500	0	0
		$[0,1,2]$	500	0	0	500	0	0	500	0	0	500	0	0	500	0	0

In Table D2, the performance of indices is quite different than in Table C1. Now, the AIC, SABIC, log-likelihood and HQ perform quite well. Notably, the performance of all indices drops somewhat when the difference in the mean ERS between groups is -1 or 0 and the number of items is small.

Table D3:

Model Fit Indices Model Selection Frequencies When the IRTree is the Generating Model

ΔERS	N_{items}	τ	AIC			BIC			SABIC			HQ			LogLik		
			MNRM	Tree	GPCM	MNRM	Tree	GPCM	MNRM	Tree	GPCM	MNRM	Tree	GPCM	MNRM	Tree	GPCM
-1	10	$[-1,0,1]$	0	500	0	0	500	0	0	500	0	0	500	0	0	500	0
		$[0,1,2]$	0	500	0	0	500	0	0	500	0	0	500	0	0	500	0
	20	$[-1,0,1]$	0	500	0	0	500	0	0	500	0	0	500	0	0	500	0
		$[0,1,2]$	0	500	0	0	500	0	0	500	0	0	500	0	0	500	0
0	10	$[-1,0,1]$	0	500	0	0	500	0	0	500	0	0	500	0	0	500	0
		$[0,1,2]$	0	500	0	0	500	0	0	500	0	0	500	0	0	500	0
	20	$[-1,0,1]$	0	500	0	0	500	0	0	500	0	0	500	0	0	500	0
		$[0,1,2]$	0	500	0	0	500	0	0	500	0	0	500	0	0	500	0

1	10	$[-1,0,1]$	0	500	0	0	500	0	0	500	0	0	500	0	0	500	0
		$[0,1,2]$	0	500	0	0	500	0	0	500	0	0	500	0	0	500	0
	20	$[-1,0,1]$	0	500	0	0	500	0	0	500	0	0	500	0	0	500	0
		$[0,1,2]$	0	500	0	0	500	0	0	500	0	0	500	0	0	500	0

Table D3 displays a very different trend than Table D2. Now, all indices achieve perfect performance.

Appendix E: Various extra conditions considered in the simulation study

Based on reviewer recommendations, we decided to include some extra conditions in the simulations. As these conditions were very similar to the main results presented in the paper, we refer the interested reader to the explanation of results given there. Note that these conditions were based on 100 rather than 500 replications to save computational time.

Table E1

Results for the MNRM Condition with N = 250 (100 replications)

Factors	ΔERS	N_{items}	$\boldsymbol{\tau}$	MNRM		IRTree		GPCM	
				μ_θ bias	σ^2 bias	μ_θ bias	σ^2 bias	μ_θ bias	σ^2 bias
MNRM	-1	10	$[-1,0,1]$	0.002	-0.008	-0.002	**-0.253**	-0.005	**-0.591**
			$[0,1,2]$	-0.001	0.003	**0.143**	**-0.254**	**0.316**	**-0.544**
		20	$[-1,0,1]$	-0.001	0.000	0.002	**-0.259**	0.011	**-0.566**
			$[0,1,2]$	0.013	0.022	**0.160**	**-0.270**	**0.334**	**-0.534**
	0	10	$[-1,0,1]$	0.011	0.011	0.009	0.011	0.008	0.010
			$[0,1,2]$	-0.013	0.023	-0.010	0.019	-0.008	0.012
		20	$[-1,0,1]$	-0.005	0.037	-0.010	0.028	-0.002	0.026
			$[0,1,2]$	0.002	-0.001	-0.003	-0.014	-0.010	-0.012
	1	10	$[-1,0,1]$	-0.013	0.030	-0.009	**0.400**	-0.004	**1.456**
			$[0,1,2]$	-0.031	0.054	**-0.217**	**0.437**	**-0.521**	**1.278**
		20	$[-1,0,1]$	0.004	0.030	-0.006	**0.435**	-0.002	**1.373**
			$[0,1,2]$	-0.005	0.014	**-0.205**	**0.397**	**-0.492**	**1.100**

Note. μ_θ bias refers to bias in the substantive trait mean in group 2, σ^2 bias refers to bias in the substantive trait variance in group 2, ΔERS refers to the difference in the ERS mean between group 1 (constant ERS mean at 0) and group 2 (-1, 0 or 1 ERS mean), N_{items} refers

to the number of items in the condition, **τ** refers to the mean item thresholds. Values substantially differing from zero are marked in bold.

Table E2

Results for the IRTree Condition with N = 250 (100 replications)

Factors	ΔERS	N_{items}	**τ**	MNRM		IRTree		GPCM	
				μ_θ bias	σ^2 bias	μ_θ bias	σ^2 bias	μ_θ bias	σ^2 bias
IRTree	-1	10	$[-1,0,1]$	0.003	**0.379**	0.001	0.032	0.001	**-0.484**
			$[0,1,2]$	**-0.180**	**0.398**	-0.001	0.027	**0.258**	**-0.453**
		20	$[-1,0,1]$	0.006	**0.359**	-0.006	0.006	-0.004	**-0.467**
			$[0,1,2]$	**-0.205**	**0.393**	-0.015	-0.004	**0.253**	**-0.447**
	0	10	$[-1,0,1]$	-0.007	0.019	-0.007	0.009	-0.002	-0.004
			$[0,1,2]$	-0.006	0.053	-0.005	0.048	0.000	0.025
		20	$[-1,0,1]$	0.007	0.016	0.001	0.014	0.005	0.018
			$[0,1,2]$	0.013	0.031	-0.003	0.016	-0.004	0.018
	1	10	$[-1,0,1]$	0.017	**-0.279**	0.015	-0.003	0.014	**0.893**
			$[0,1,2]$	**0.192**	**-0.290**	0.010	0.009	**-0.345**	**0.865**
		20	$[-1,0,1]$	0.006	**-0.289**	-0.008	0.002	-0.009	**0.813**
			$[0,1,2]$	**0.210**	**-0.273**	0.008	0.032	**-0.310**	**0.750**

Note. Notation is as described above for the MNRM table.

Table E3

Results for the MNRM Condition with N = 1000 (100 replications)

Factors	ΔERS	N_{items}	$\boldsymbol{\tau}$	MNRM		IRTree		GPCM	
				μ_θ bias	σ^2 bias	μ_θ bias	σ^2 bias	μ_θ bias	σ^2 bias
MNRM	-1	10	$[-1,0,1]$	0.003	0.010	0.002	**-0.246**	0.000	**-0.591**
			[0,1,2]	-0.007	0.017	**0.140**	**-0.248**	**0.319**	**-0.540**
		20	$[-1,0,1]$	-0.001	0.006	-0.001	**-0.271**	0.002	**-0.583**
			[0,1,2]	-0.013	0.011	**0.140**	**-0.269**	**0.312**	**-0.520**
	0	10	$[-1,0,1]$	-0.004	-0.006	-0.005	-0.006	-0.005	-0.005
			[0,1,2]	-0.004	0.013	-0.007	0.018	-0.011	0.024
		20	$[-1,0,1]$	0.004	0.026	0.001	0.015	0.003	0.011
			[0,1,2]	0.000	0.023	0.004	0.018	0.005	0.004
	1	10	$[-1,0,1]$	0.000	0.006	0.000	**0.366**	-0.001	**1.420**
			[0,1,2]	0.004	0.004	**-0.186**	**0.382**	**-0.500**	**1.229**
		20	$[-1,0,1]$	0.009	0.047	0.005	**0.423**	0.002	**1.337**
			[0,1,2]	-0.006	0.034	**-0.196**	**0.416**	**-0.468**	**1.092**

Note. μ_θ bias refers to bias in the substantive trait mean in group 2, σ^2 bias refers to bias in the substantive trait variance in group 2, ΔERS refers to the difference in the ERS mean between group 1 (constant ERS mean at 0) and group 2 (-1, 0 or 1 ERS mean), N_{items} refers to the number of items in the condition, $\boldsymbol{\tau}$ refers to the mean item thresholds. Values substantially differing from zero are marked in bold.

Table E4

Results for the IRTree Condition with N = 1000 (100 replications)

Factors	ΔERS	N_{items}	$\boldsymbol{\tau}$	MNRM		IRTree		GPCM	
				μ_θ bias	σ^2 bias	μ_θ bias	σ^2 bias	μ_θ bias	σ^2 bias
IRTree	-1	10	$[-1,0,1]$	0.002	**0.339**	0.004	-0.002	0.006	**-0.498**
			$[0,1,2]$	**-0.185**	**0.383**	-0.002	0.013	**0.260**	**-0.457**
		20	$[-1,0,1]$	0.005	**0.368**	0.005	-0.003	0.004	**-0.476**
			$[0,1,2]$	**-0.184**	**0.408**	0.004	0.011	**0.262**	**-0.438**
	0	10	$[-1,0,1]$	0.003	0.004	0.002	0.003	0.005	0.003
			$[0,1,2]$	0.005	-0.015	0.004	-0.015	0.002	-0.012
		20	$[-1,0,1]$	-0.006	0.022	-0.007	0.011	-0.007	0.009
			$[0,1,2]$	-0.004	0.015	0.000	0.007	-0.003	0.006
	1	10	$[-1,0,1]$	-0.005	**-0.280**	-0.005	-0.004	-0.003	**0.880**
			$[0,1,2]$	**0.171**	**-0.289**	-0.010	0.013	**-0.351**	**0.853**
		20	$[-1,0,1]$	-0.008	**-0.271**	-0.006	0.011	-0.004	**0.797**
			$[0,1,2]$	**0.204**	**-0.286**	0.008	0.007	**-0.308**	**0.719**

Note. Notation is as described above for the MNRM table.

Table E5

Results for the MNRM Condition with m-values based on the normal distribution quantiles

Factors	ΔERS	N_{items}	$\boldsymbol{\tau}$	MNRM		IRTree		GPCM	
				μ_θ bias	σ^2 bias	μ_θ bias	σ^2 bias	μ_θ bias	σ^2 bias
MNRM	-1	10	$[-1,0,1]$	-0.004	0.009	-0.006	**-0.259**	-0.007	**-0.597**
			[0,1,2]	0.016	0.006	**0.165**	**-0.266**	**0.335**	**-0.549**
		20	$[-1,0,1]$	-0.003	-0.003	-0.005	**-0.282**	0.002	**-0.578**
			[0,1,2]	0.004	0.019	**0.157**	**-0.281**	**0.323**	**-0.531**
	0	10	$[-1,0,1]$	0.000	0.011	-0.001	0.014	0.000	0.016
			[0,1,2]	0.015	0.015	0.011	0.019	0.003	0.031
		20	$[-1,0,1]$	0.000	0.018	-0.001	0.009	0.003	0.005
			[0,1,2]	-0.008	0.011	-0.006	0.011	-0.004	0.005
	1	10	$[-1,0,1]$	0.001	0.065	0.003	**0.460**	0.004	**1.550**
			[0,1,2]	-0.014	0.032	**-0.206**	**0.427**	**-0.507**	**1.257**
		20	$[-1,0,1]$	-0.007	0.033	-0.010	**0.442**	-0.012	**1.387**
			[0,1,2]	0.006	0.044	**-0.209**	**0.441**	**-0.495**	**1.143**

Note. μ_θ bias refers to bias in the substantive trait mean in group 2, σ^2 bias refers to bias in the substantive trait variance in group 2, ΔERS refers to the difference in the ERS mean between group 1 (constant ERS mean at 0) and group 2 (-1, 0 or 1 ERS mean), N_{items} refers to the number of items in the condition, $\boldsymbol{\tau}$ refers to the mean item thresholds. Values substantially differing from zero are marked in bold.

Instead of the uniformly distributed m-values used in the main paper simulation study, the m-values here were based on a normal distribution with a mean of zero and a standard

deviation of 0.3. 10 or 20 (depending on the number of items) equidistant numbers were drawn from 0.05 to 0.95. The m-values were then calculated as the quantiles at these points using the qnorm function. This was done to create non-equidistant m-values, with most m-values lying close to the mean of the distribution (zero), while the m-values remain symmetrical on both sides and cover approximately the same area as the uniform m-values used in the main simulation study.

Table E6

Results for the IRTree Condition with m-values based on the normal distribution quantiles

Factors	ΔERS	N_{items}	τ	MNRM		IRTree		GPCM	
				μ_θ bias	σ^2 bias	μ_θ bias	σ^2 bias	μ_θ bias	σ^2 bias
IRTree	-1	10	$[-1,0,1]$	-0.005	**0.351**	-0.004	0.000	0.000	**-0.491**
			$[0,1,2]$	**-0.181**	**0.359**	0.004	-0.018	**0.260**	**-0.477**
		20	$[-1,0,1]$	0.013	**0.415**	0.011	0.015	0.009	**-0.473**
			$[0,1,2]$	**-0.202**	**0.424**	-0.008	0.010	**0.251**	**-0.439**
	0	10	$[-1,0,1]$	0.009	0.003	0.006	0.007	0.002	0.013
			$[0,1,2]$	-0.005	0.009	-0.003	0.009	0.002	0.003
		20	$[-1,0,1]$	0.002	0.016	-0.004	0.006	0.000	0.003
			$[0,1,2]$	-0.008	0.036	-0.011	0.026	-0.010	0.018
	1	10	$[-1,0,1]$	0.011	**-0.273**	0.008	0.023	0.008	**0.952**
			$[0,1,2]$	**0.194**	**-0.293**	0.005	0.018	**-0.347**	**0.882**
		20	$[-1,0,1]$	0.008	**-0.278**	-0.006	0.021	-0.007	**0.832**
			$[0,1,2]$	**0.220**	**-0.303**	0.008	-0.007	**-0.319**	**0.726**

Note. Notation is as described above for the MNRM table.

Appendix F: MAE and RMSE for the simulation conditions in the main paper

Based on reviewer recommendations, we decided to calculate the MAE and RSME for every simulation condition mentioned in the main paper. Results were again similar to the main simulation study, but this time give more insight into the individual level rather than just the substantive trait mean and variance.

Table F1

MAE and RMSE for the control condition for the GPCM condition

Factors	N_{items}	$\boldsymbol{\tau}$	MNRM		IRTree		GPCM	
			MAE	$RMSE$	MAE	$RMSE$	MAE	$RMSE$
GPCM	10	$[-1,0,1]$	0.246	0.314	0.247	0.314	0.246	0.313
		$[0,1,2]$	0.280	0.363	0.280	0.363	0.279	0.362
	20	$[-1,0,1]$	0.181	0.232	0.182	0.233	0.180	0.231
		$[0,1,2]$	0.279	0.362	0.279	0.362	0.278	0.361

Note. MAE refers to the mean absolute bias, RMSE refers to the root of the mean squared error, N_{items} refers to the number of items in the condition, and $\boldsymbol{\tau}$ refers to the mean item thresholds.

As can be seen in Table F1, the MAE and RMSE for every model are very similar when the GPCM generates the data.

Table F2

MAE and RMSE for the MNRM Condition

Factors	ΔERS	N_{items}	$\boldsymbol{\tau}$	MNRM		IRTree		GPCM	
				MAE	$RMSE$	MAE	$RMSE$	MAE	$RMSE$
MNRM	-1	10	$[-1,0,1]$	0.318	0.411	0.325	0.422	0.391	0.512
			$[0,1,2]$	0.378	0.491	0.391	0.512	0.475	0.621
		20	$[-1,0,1]$	0.254	0.335	0.268	0.357	0.358	0.478
			$[0,1,2]$	0.315	0.421	0.337	0.452	0.448	0.592
	0	10	$[-1,0,1]$	0.308	0.401	0.313	0.407	0.357	0.467
			$[0,1,2]$	0.374	0.491	0.381	0.499	0.432	0.563
		20	$[-1,0,1]$	0.249	0.332	0.259	0.347	0.330	0.441
			$[0,1,2]$	0.316	0.426	0.329	0.442	0.409	0.540
	1	10	$[-1,0,1]$	0.298	0.393	0.318	0.417	0.445	0.609
			$[0,1,2]$	0.373	0.495	0.407	0.536	0.563	0.762
		20	$[-1,0,1]$	0.245	0.331	0.272	0.369	0.411	0.580
			$[0,1,2]$	0.319	0.435	0.361	0.490	0.526	0.723

As can be seen in Table F2, the MNRM always achieves the lowest MAE and RMSE when it is the data-generating model. In addition, the MAE and RMSE decrease in the 20-item condition compared to the 10-item condition. Threshold shifts also lead to an increase in the MAE and RMSE, as the item difficulties are less matched to the ability level in the population, leading to decreased test information. Other models follow the same trend.

Between models, we can see that the MNRM achieves superior MAE and RMSE values over the IRTree model. The difference in MAE is generally quite small (~0.1) between the models, except in the condition where the mean ERS is positive. The same trend is visible

in the RMSE. Note that this reflects the larger differences between the estimated substantive trait mean and variance between the models we found in the main simulation study .

Differences between the GPCM and MNRM are substantially larger than those between the IRTree and MNRM, which was also found in the simulation study. Even when the mean ERS is zero, the GPCM is inferior to both the MNRM and IRTree in terms of MAE and RMSE. Interestingly, this is not reflected in the group level results in the main paper. As the MNRM and IRTree perform similar to the GPCM when there is in fact no ERS present, but the GPCM performs far worse when ERS is in fact present, it may be preferable to model using an ERS regardless of the presence of ERS. Nevertheless, results may look different for conditions we did not consider in this paper, so researchers should be cautious in taking this approach. Similar to the IRTree, the GPCM performs especially bad in terms of MAE and RMSE when the mean ERS difference between groups is positive.

Table F3

MAE and RMSE for the IRTree Condition

Factors	ΔERS	N_{items}	$\boldsymbol{\tau}$	MNRM		IRTree		GPCM	
				MAE	$RMSE$	MAE	$RMSE$	MAE	$RMSE$
IRTree	-1	10	$[-1,0,1]$	0.300	0.388	0.283	0.368	0.336	0.445
			$[0,1,2]$	0.368	0.478	0.336	0.441	0.404	0.536
		20	$[-1,0,1]$	0.251	0.330	0.221	0.294	0.295	0.400
			$[0,1,2]$	0.323	0.428	0.275	0.372	0.367	0.496
	0	10	$[-1,0,1]$	0.285	0.372	0.280	0.365	0.312	0.409
			$[0,1,2]$	0.344	0.453	0.335	0.442	0.370	0.486
		20	$[-1,0,1]$	0.233	0.310	0.218	0.291	0.270	0.363

Factors	ΔERS	N_{items}	τ	MNRM		IRTree		GPCM	
				MAE	$RMSE$	MAE	$RMSE$	MAE	$RMSE$
			[0,1,2]	0.292	0.393	0.273	0.371	0.333	0.447
	1	10	[−1,0,1]	0.290	0.386	0.279	0.365	0.360	0.485
			[0,1,2]	0.361	0.488	0.338	0.448	0.445	0.602
		20	[−1,0,1]	0.241	0.328	0.218	0.292	0.313	0.434
			[0,1,2]	0.315	0.435	0.275	0.376	0.400	0.552

Table F3 presents the results for the IRTree condition. These are similar to results shown for the MNRM condition. Once again, the IRTree always performs best, especially when the number of items is higher and no threshold shift is present. The MNRM is quite close to the IRTree when there is no ERS difference between groups, but drops in performance when groups differ in mean ERS. Note that the asymmetry in positive and negative ERS performance is less strongly present in the IRTree condition than in the MNRM condition, which is a results also reflected in the main simulation results. Once again, the GPCM shows an inferior performance to the ERS models in all conditions.
